# Muraymycin nucleoside-peptide antibiotics: uridine-derived natural products as lead structures for the development of novel antibacterial agents

**DOI:** 10.3762/bjoc.12.77

**Published:** 2016-04-22

**Authors:** Daniel Wiegmann, Stefan Koppermann, Marius Wirth, Giuliana Niro, Kristin Leyerer, Christian Ducho

**Affiliations:** 1 Department of Pharmacy, Pharmaceutical and Medicinal Chemistry, Saarland University, Campus C2 3, 66123 Saarbruecken, Germany

**Keywords:** antibiotics, natural products, nucleosides, peptides, structure–activity relationship

## Abstract

Muraymycins are a promising class of antimicrobial natural products. These uridine-derived nucleoside-peptide antibiotics inhibit the bacterial membrane protein translocase I (MraY), a key enzyme in the intracellular part of peptidoglycan biosynthesis. This review describes the structures of naturally occurring muraymycins, their mode of action, synthetic access to muraymycins and their analogues, some structure–activity relationship (SAR) studies and first insights into muraymycin biosynthesis. It therefore provides an overview on the current state of research, as well as an outlook on possible future developments in this field.

## Introduction

The treatment of infectious diseases caused by bacteria is a severe issue. With multiresistant bacterial strains rendering well-established therapeutic procedures ineffective, the exploration of novel antimicrobial agents is of growing significance. The discovery of penicillin [1] and the proof of its in vivo efficacy [2] marked the starting point for the research on antibacterial drugs during the so-called "golden age" of antibiotics. Despite the early occurrence of first resistances [3–5], an innovation gap followed from the 1960s onwards, during which only few antibiotics were introduced into the market. Most of them were modifications of established substances already in clinical use. Current and future developments will have to consider these improved 2nd and 3rd generation antibiotics [6] alongside the search for completely unknown structures. For such novel agents, natural products appear to be a promising source [7–9].

Bacteria deploy different mechanisms to escape the toxic effect of an antibacterial drug [10–12]. These include the structural modification and degradation of a drug, as it is reported for aminoglycoside-modifying proteins [13], and alteration of the drug target, as can be found in macrolide-resistant bacteria that contain mutations in the bacterial ribosome [14]. Further mechanisms are an increased efflux [15] and a change in permeability of the cell wall [16–17]. Due to the evolutionary pressure exerted by antibiotics, bacteria featuring the aforementioned mutations survive, proliferate and may even develop resistances against multiple drug classes. Excessive application of antibiotics fuels the emergence of multiresistant strains such as hospital and community-associated methicillin-resistant *Staphylococcus aureus* (MRSA) [18–19] and vancomycin-resistant *Enterococcus* (VRE) [20]. This development raises the demand for antibiotics exploiting yet unused modes of action. Potential targets within bacteria include peptidoglycan biosynthesis, protein biosynthesis, DNA and RNA replication and folate metabolism [21].

Promising candidates meeting the requirements for new drugs are nucleoside antibiotics, i.e., uridine-derived compounds that address the enzyme translocase I (MraY) as a novel target, thereby interfering with a membrane-associated intracellular step of peptidoglycan biosynthesis. This review will focus on muraymycins as a subclass of nucleoside antibiotics, covering their mode of action, synthetic approaches as well as SAR studies on several derivatives. Furthermore, first insights into the biosynthesis of these *Streptomyces*-produced secondary metabolites will be discussed.

## Review

### Structures of naturally occurring muraymycins

**Figure 1:**
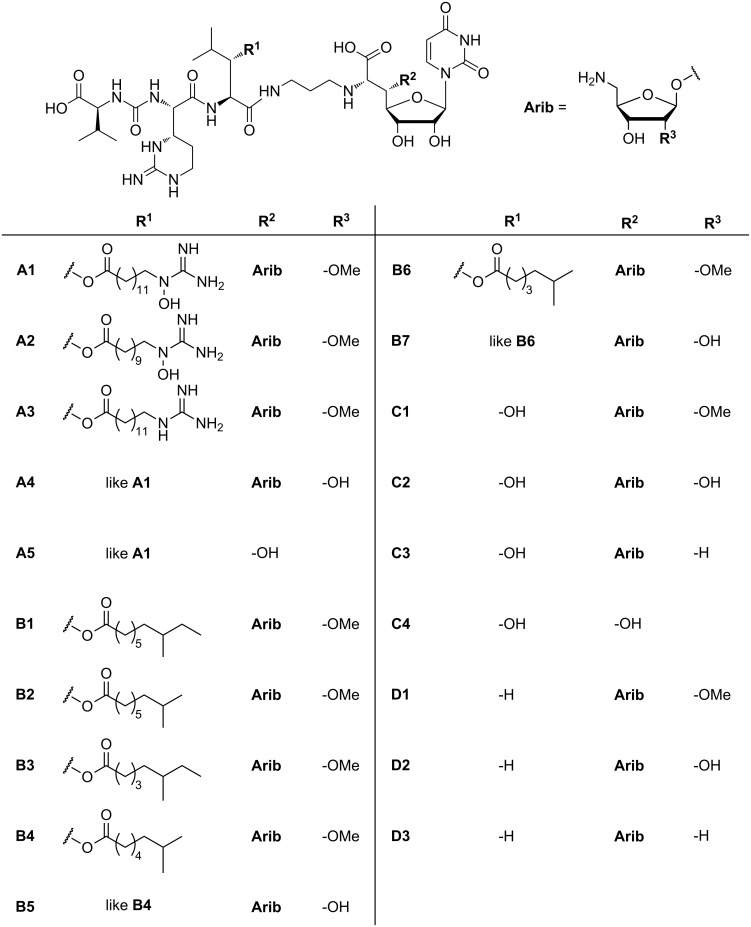
Structures of the naturally occurring muraymycins isolated by McDonald et al. [22].

The muraymycins were first isolated in 2002 from a broth of a *Streptomyces* sp. [22]. McDonald et al. discovered and characterised 19 naturally occurring muraymycins (Figure 1). These compounds belong to the family of nucleoside antibiotics which have a uridine-derived core structure in common. Their antibiotic potency is based on the inhibition of MraY, thereby blocking a membrane-associated intracellular step of bacterial cell-wall biosynthesis. The structure elucidation was carried out using one- and two-dimensional NMR experiments as well as FT mass spectrometry [22].

Muraymycins have a glycyl-uridine motif, which is connected via an aminopropyl linker to a urea peptide moiety consisting of L-leucine or L-hydroxyleucine, L-epicapreomycidine (a non-proteinogenic cyclic arginine derivative) and L-valine. The uridine structure is glycosylated in its 5'-position with an aminoribose unit and in some cases a lipophilic side chain is attached to the hydroxyleucine residue. The 19 compounds are divided into four different series (A–D) which mainly vary in the leucine residue and the lipophilic side chain or the amino sugar (Figure 1). The aminoribose is missing in muraymycins A5 and C4, which may eventually be hydrolysis products. The series A and B have lipophilic side chains with varying chain lengths, which are either ω-functionalised with a guanidino or hydroxyguanidino-function in case of series A or unfunctionalised but terminally branched in case of series B. Muraymycins of series C contain unfunctionalised L-hydroxyleucine while in series D proteinogenic L-leucine occurs instead.

Muraymycin A1 is one of the most active members of this family and shows good activity mainly against Gram-positive (*Staphylococcus* MIC: 2–16 μg/mL, *Enterococcus* MIC: 16–64 µg/mL) but also a few Gram-negative bacteria (*E. coli* MIC: down to 0.03 μg/mL). Since the activity against wild-type *E. coli* is clearly lower (MIC > 128 μg/mL) [22], it is assumed that this might be an effect resulting from low membrane permeability.

**Figure 2:**
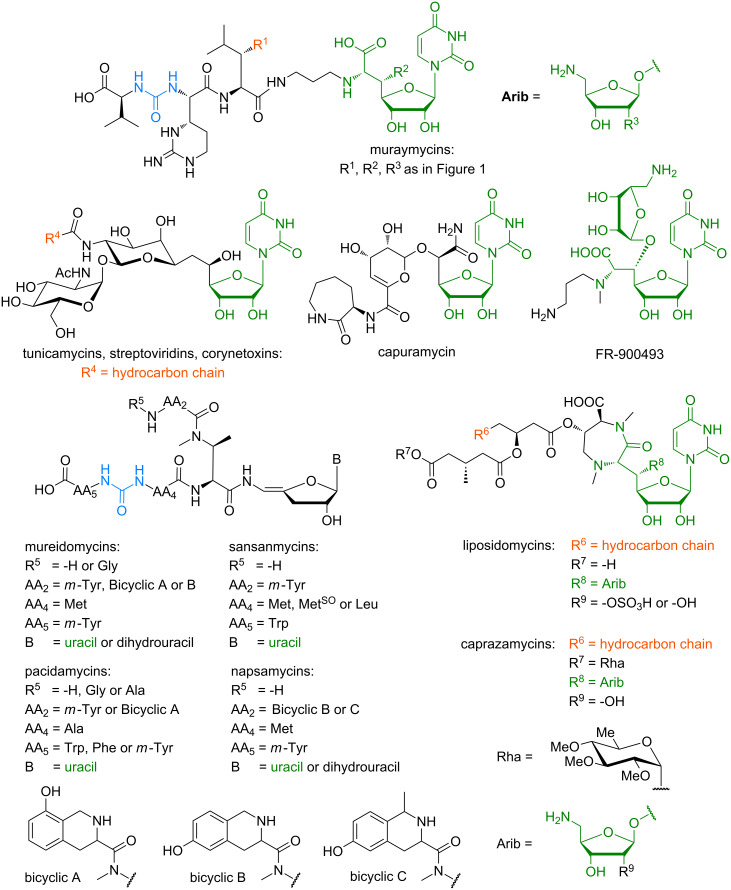
Structures of selected classes of nucleoside antibiotics. Similarities to the muraymycins are highlighted in different colours.

There are other naturally occurring nucleoside antibiotics which address the same biological target, thereby inhibiting peptidoglycan biosynthesis. Figure 2 shows the structures of selected other classes of nucleoside antibiotics, with structural similarities being highlighted. A broad overview of antimicrobial nucleoside antibiotics blocking peptidoglycan biosynthesis is given by Bugg et al. in two review articles [23–24] and by Ichikawa et al. in a recent review [25].

Representing the first discovered nucleoside antibiotics, the tunicamycins were isolated in 1971 from *Streptomyces lysosuperficus* nov. sp. by Takatsuki and Tamura et al. [26–28]. They contain a uridine moiety, two *O*-glycosidically linked sugars, the so-called tunicamine and a fatty acid moiety, which typically is terminally branched and unsaturated. Two closely related nucleoside antibiotics were isolated later on and named streptoviridins (isolated in 1975 from *Streptomyces griseoflavus* subsp. *thuringiensis* [29–31]) and corynetoxins (isolated in 1981 from *Corynebacterium rathayi* [32]). These classes have merely the uracil nucleoside core structure in common with the muraymycins and the terminally branched lipophilic side chain resembles the acyl moiety in muraymycins of group B.

Capuramycin, a nucleoside antibiotic isolated in 1986 from *Streptomyces griseus*, shares the uracil-derived nucleoside moiety with the muraymycins [33–34]. The antibiotic FR-900493, which is structurally closely related to muraymycins, was isolated from *Bacillus cereus* and characterised in 1990 [35]. In comparison to the muraymycins, only the urea peptide moiety and the lipopeptidyl motif are absent.

The mureidomycins [36–38] and pacidamycins [39–41], both reported in 1989, the napsamycins (1994) [42] and the sansanmycins (2007) [43–44] are structurally closely related. They consist of a 3'-deoxyuridine unit with a unique enamide linkage and the non-proteinogenic *N*-methyl-2,3-diaminobutyric acid, which branches into two peptide moieties. They differ in the amino acid residues AA_2_, AA_4_ and AA_5_, with AA_2_ and AA_5_ being aromatic in all four classes. The amino acid residue AA_4_ is either methionine for mureidomycins, napsamycins and sansanmycins or alanine in case of pacidamycins. Remarkably, these natural products share a urea peptide motif with the muraymycins. They are mainly active against Gram-negative bacteria, which is a noteworthy difference to the muraymycins and other related nucleoside antibiotics.

The liposidomycins (isolated in 1985) [45] and the related caprazamycins (isolated in 2003) [46–47] have a unique diazepanone ring, and in case of the caprazamycins a permethylated rhamnose residue. They resemble the muraymycins in their uridine-derived core structure, which is also glycosylated in 5'-position with an aminoribose unit, and they contain a fatty acid moiety as well. Caprazamycins also display noteworthy antimicrobial activity against *M. tuberculosis* as well as most Gram-positive bacteria (Table 1) [46,48].

**Table 1:** Comparison of the antimicrobial activities of selected representative compounds of different classes of nucleoside antibiotics against selected bacterial species.^a^

	Gram-positive			Gram-negative	
	
	*S. aureus*	*B. subtilis*	*M. smegmatis*	*E. coli*	*P. aeruginosa*

Muraymycin A1	++	n.r.	n.r.	++/+^b^	+/−
Tunicamycin	−	++	−	−	−
Capuramycin	−	−	++	−	−
FR-900493	++	++	n.r.	n.r.	n.r.
Mureidomycin C	−	n.r.	n.r.	−	++
Caprazamycin B	++	++/+	++	−	++
Liposidomycin A	−	−	n.r.^c^	−	n.r.

^a^++: good activity (MIC < 10 μg/mL), +: moderately active (10 μg/mL < MIC < 32 μg/mL), −: no notable activity (MIC > 32 μg/mL), n.r.: not reported. ^b^Not active against wild-type *E. coli*. ^c^Active against *M. phlei*.

All aforementioned nucleoside antibiotics address the same biological target and most likely have the same mode of action by inhibiting MraY (see below), but their in vitro activity differs significantly. It is important to notice that a comprehensive comparison of minimum inhibitory concentrations (MIC values) is difficult because naturally occurring nucleoside antibiotics have been tested against different bacterial strains. However, synthetic analogues of the nucleoside antibiotics listed in Table 1 have been tested against some of the listed bacterial species. It can therefore be assumed that the parent natural products display similar activities even though there are no data available. Furthermore, the activity of a compound against different strains of a bacterial species can vary. Nonetheless, there are certain trends and differences that can be observed. Muraymycin A1 is mainly active against Gram-positive bacteria such as *S. aureus* or *E. faecalis*, but also against some Gram-negative *E. coli* strains [49]. Tunicamycin, capuramycin and FR-900493 only show antimicrobial activity against Gram-positive strains. For mureidomycin C (R^5^ = Gly, AA_2_ = AA_5_ = *m*-Tyr, AA_4_ = Met, B = uracil, see Figure 2) as a representative compound, no activity against Gram-positive bacteria was observed, but it displayed pronounced antibacterial activity against *P. aeruginosa*. This remarkable finding distinguishes the mureidomycins, pacidamycins, sansanmycins and napsamycins from other nucleoside antibiotics. On the other hand, caprazamycin B shows good activity against Gram-positive bacteria, *Pseudomonas* and *M. tuberculosis* [48]. The related liposidomycins display good activity against *M. phlei*, while they are not active against a range of other bacteria [45].

### Mode of action

**Figure 3:**
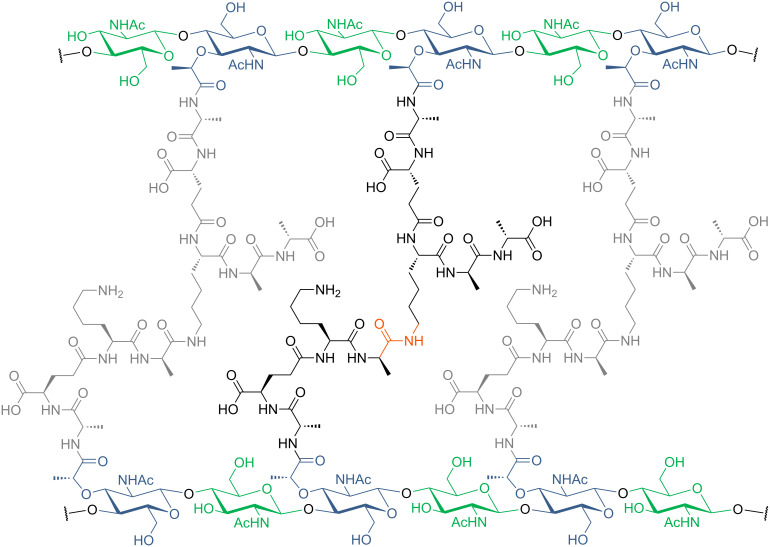
Structure of peptidoglycan. Long chains of glycosides (alternating Glc*N*Ac (green) and Mur*N*Ac (blue)) are cross-linked through the Mur*N*Ac peptide chain. The exact composition of the peptide chain varies among different bacterial species.

To develop an effective antibiotic one needs to choose a target that is essential for bacterial survival or growth and offers selectivity to strike only bacterial cells (without cytotoxicity to human cells). There are mainly four classical target processes for antibiotics: bacterial cell wall biosynthesis, bacterial protein biosynthesis, DNA replication and folate metabolism [21]. Novel approaches that differ from these established modes of action are under investigation, but many new compounds in development still address bacterial cell wall biosynthesis. They are accompanied by a rich variety of prominent antibiotics in clinical use such as the penicillins [23,50–51]. All bacteria, i.e., Gram-positive and Gram-negative congeners, have a cell wall as part of their cell envelope. While its thickness differs among bacteria – Gram-positive strains usually have a thicker cell wall relative to Gram-negative ones – the principle molecular structure remains identical: Bacterial cell walls consist of peptidoglycan, a heteropolymer with long chains of alternating units of *N*-acetylmuramic acid (Mur*N*Ac) and *N*-acetylglucosamine (Glc*N*Ac) that are cross-linked through peptide chains attached to the muramic acid sugar (Figure 3) [52].

**Figure 4:**
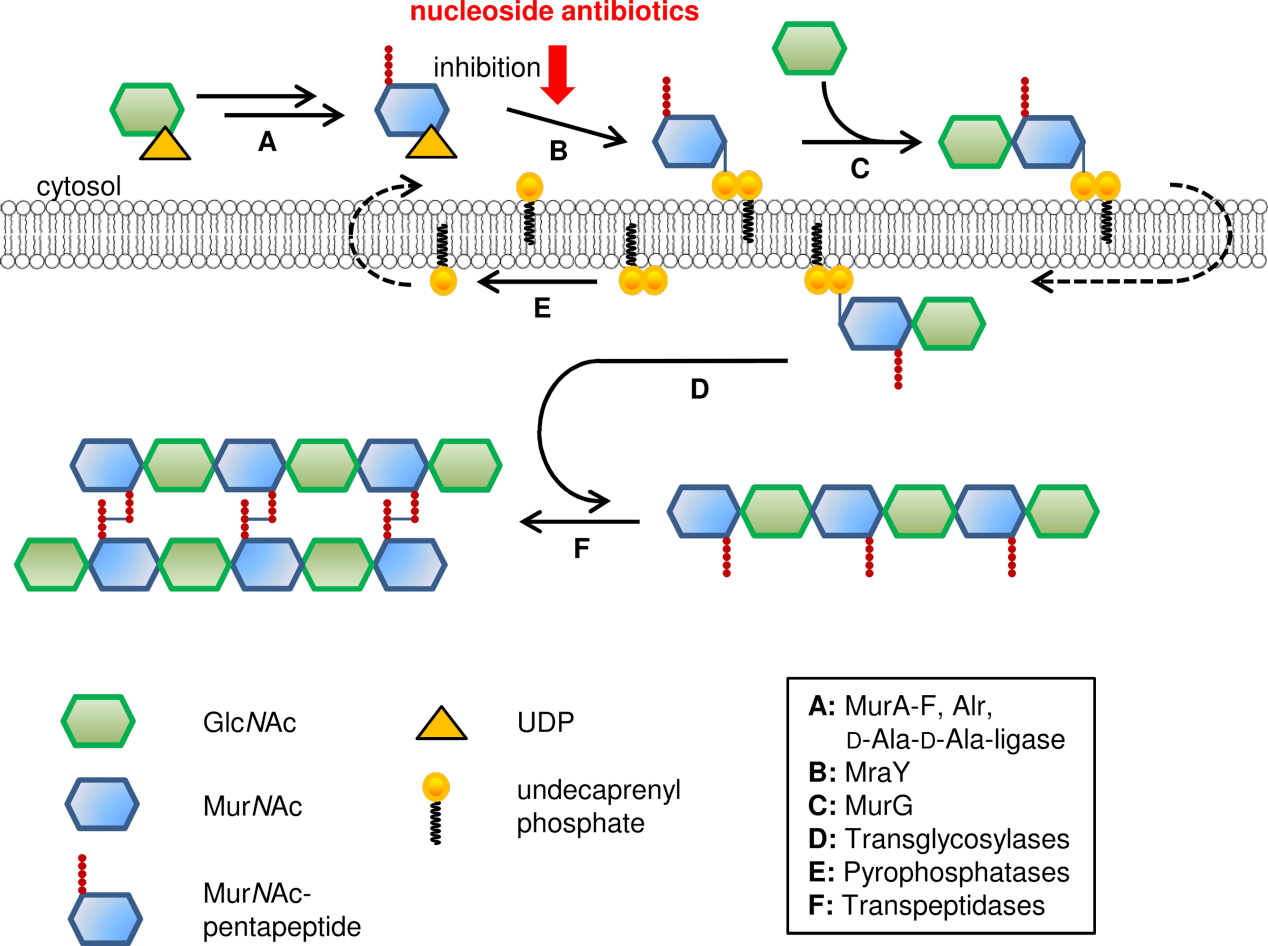
Schematic representation of bacterial cell wall biosynthesis.

The biosynthesis of peptidoglycan is illustrated in Figure 4 and has been described in detail in several reviews (e.g., [51,53–57]). It can be divided into three parts: first, the formation of the monomeric building blocks in the cytosol (Figure 4, step A); second, the membrane-bound steps with the attachment to the lipid linker, transformation to a disaccharide and transport to the extracellular side of the membrane (Figure 4, steps B, C); finally, polymerisation to long oligosaccharide chains and cross-linking occur (Figure 4, steps D, F).

In the cytosol, uridine diphosphate-*N*-acetylglucosamine (UDP-Glc*N*Ac), that is formed from fructose-6-phosphate in four steps, is transformed into UDP-Mur*N*Ac-pentapeptide in a number of enzyme-catalysed reactions (Figure 4, step A). The exact composition of the peptide chain varies in different organisms. Examples given in Figure 3 are frequently occurring ones and a more comprehensive list has been reported elsewhere [52].

The membrane-associated steps commence with the transfer of UDP-Mur*N*Ac-pentapeptide to the lipid carrier undecaprenyl phosphate, catalysed by translocase I (MraY), to give lipid I (Figure 4, product of step B). The glycosyltransferase MurG attaches a Glc*N*Ac sugar to furnish lipid II (Figure 4, product of step C). This building block is then transported to the extracellular side of the membrane. It is speculated that there might be some kind of 'flippase' involved but this particular step is still unclear and requires further investigation [55]. On the extracellular side of the membrane, the building blocks are connected by transglycosylases to form long chains (Figure 4, step D) and then are cross-linked by transpeptidases (Figure 4, step E). Both enzymes are members of the family of penicillin-binding proteins [23].

As mentioned above, there are many antibiotics in clinical use that target at least one step of bacterial cell wall biosynthesis. Prominent examples besides penicillins are cephalosporins, cycloserine, vancomycin, fosfomycin and daptomycin [9]. All of them (except fosfomycin and cycloserine) inhibit late, extracellular steps of cell wall formation. Thus, there are still many steps not addressed by clinically used drugs, which implies that cell wall biosynthesis still offers promising novel targets for the development of antibiotics with new modes of action. Muraymycins and other nucleoside antibiotics target translocase I (MraY) that represents such a potential novel molecular target [22].

Overexpression of the *mraY* gene, identified in an *mra* (murein region A) cluster, led to an increase of UDP-*N*-acetylmuramoyl-pentapeptide: undecaprenyl phosphate phospho-*N*-acetylmuramoyl-pentapeptide transferase activity [58]. Gene knockout experiments revealed the MraY-catalysed reaction in cell wall biosynthesis to be an essential process for bacterial viability and growth [59–63].

**Figure 5:**
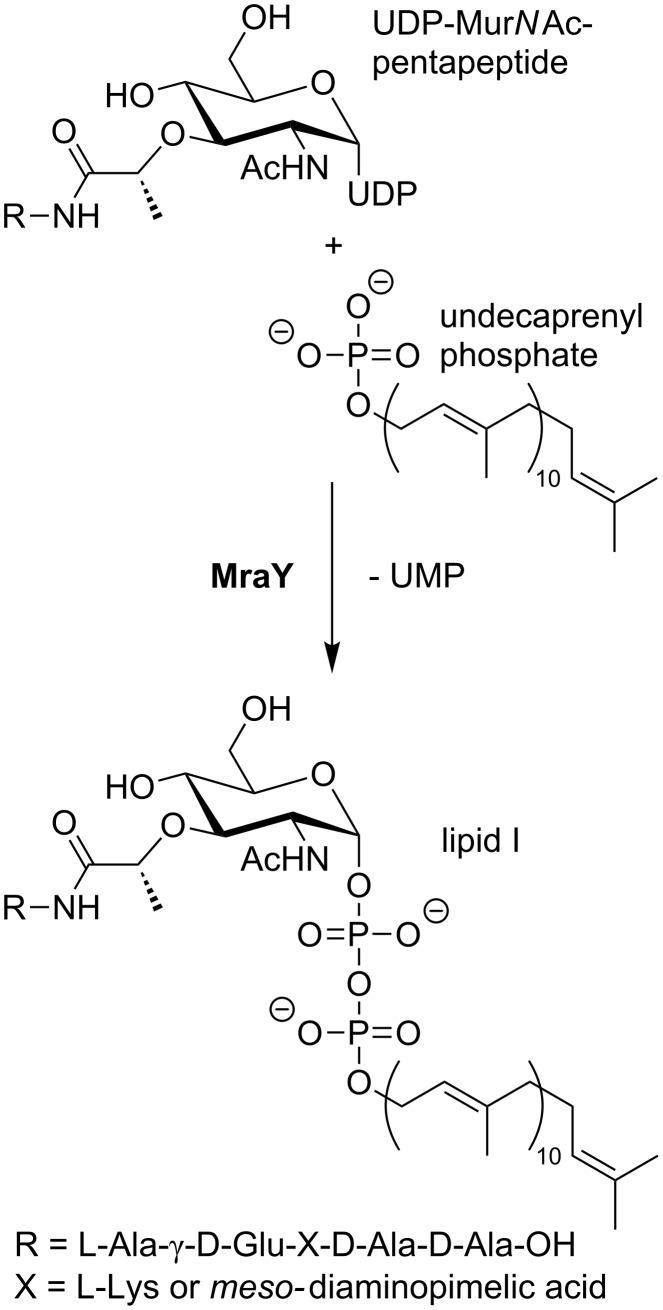
Translocase I (MraY) catalyses the reaction of UDP-Mur*N*Ac-pentapeptide with undecaprenyl phosphate towards lipid I.

**Figure 6:**
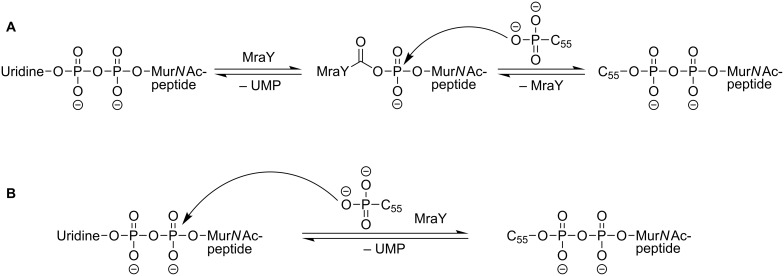
Proposed mechanisms for the MraY-catalysed reaction. A: Two-step mechanism postulated by Heydanek et al. [66]; B: one-step mechanism postulated by Bouhss et al. [69].

The chemical transformation catalysed by MraY is shown in Figure 5. The cytosolic precursor UDP-Mur*N*Ac-pentapeptide is linked to undecaprenyl phosphate, a C_55_-isoprenoid lipid carrier that is located in the cellular membrane. With concomitant release of uridine monophosphate (UMP), this furnishes a diphosphate linkage between the two substrates. The reaction is reversible and MraY accelerates the adjustment of the equilibrium state. Whereas this reaction was known for a long time [64–65], the structure of the MraY protein remained unclear. The mechanism of the MraY-catalysed reaction was investigated by kinetic studies by Heydanek, Neuhaus et al. in the 1960s. They proposed a two-step mechanism for lipid I formation that was later revised (Figure 6A) [55,66–69].

The identification of the *mraY* gene [58] facilitated the alignment of MraY homologue sequences by van Heijenoort et al. and resulted in a two-dimensional topology model of MraY from *E. coli*, among others [70]. Bugg et al. identified three conserved residues with nucleophilic side chains within the superfamily of polyisoprenyl-phosphate *N*-acetyl hexosamine 1-phosphate transferases (PNPT). Mutation of these three aspartate residues (D115, D116 and D267 in the *E. coli* protein) resulted in a complete loss of catalytic activity. This led to a proposed model for the active site of MraY in accordance with previous findings [66]: D115 and D116 bind a Mg^2+^-cofactor, UDP-Mur*N*Ac-pentapeptide also binds the Mg^2+^-cofactor and D267 acts as a nucleophile within the proposed two-step mechanism (Figure 6) [68]. In a study with purified MraY from *B. subtilis*, Bouhss et al. found small remaining activity in the D231N mutant (corresponding to D267 in MraY from *E. coli*). They assumed that this would contradict the two-step mechanism as a nucleophilic residue is essential for the previously proposed mechanism. They found D98 to be crucial for activity and proposed its role to deprotonate undecaprenyl phosphate. This was speculated to be followed by a one-step nucleophilic attack of the C_55_-alkyl phosphate at the UDP-Mur*N*Ac-pentapeptide (Figure 6B) [69].

In 2013, Lee et al. reported an X-ray crystal structure (3.3 Å resolution) of MraY from *Aquifex aeolicus* (MraY_AA_) as the first structure of a member of the PNPT superfamily. MraY_AA_ crystallised as a dimer and additional experiments showed that it also exists as a dimer in detergent micelles and membranes [71]. The previously proposed models are in agreement with the solved structure showing ten transmembrane helices and five cytoplasmic loops. The authors identified a cleft at the cytoplasmic side of the membrane that showed the highest conservation in sequence mapping. Furthermore, it is also the region where most of the previously identified, functionally important residues [69] are located [71]. The location and binding mode of the Mg^2+^ ion in the crystal does not support the proposed model for a two-step mechanism [68]. In experiments with Mn^2+^ exchange no interaction of the metal with D117 and D118 could be detected. Surface calculation of MraY_AA_ showed an inverted U-shaped groove that could harbour the undecaprenyl phosphate co-substrate. The locations of this groove, the Mg^2+^ and D265 do at least not contradict the proposed one-step mechanism. Nevertheless, there is still a need for further studies to fully understand the MraY-catalysed reaction at the molecular level [71].

In the context of a different MraY inhibitor, i.e., lysis protein E from bacteriophage 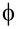
X174, Bugg et al. reported a different site of inhibition in pronounced distance to the proposed active site. It has been demonstrated before that mutation of phenylalanine 288 (F288L) in helix 9 of MraY caused resistance against lysis protein E [72–73]. An interaction between F288 and glutamic acid 287 (E287) with the peptide motif arginine-tryptophan-x-x-tryptophan (RWxxW, x represents an arbitrary amino acid) was found. Mutants F288L and E287A showed reduced or no detectable enzyme inhibition, thus indicating a secondary binding site for potential MraY inhibitors. Nevertheless, it remains unclear how binding at helix 9 can inhibit MraY function and further studies are probably inevitable [74].

In order to investigate the biological potencies of MraY inhibitors such as the muraymycins, in vitro assay systems are needed. A widely used and universal method to evaluate the in vitro activity of potential agents against certain bacteria is the determination of minimum inhibitory concentrations (MIC). MICs are defined as the lowest concentration at which a potential antimicrobial agent inhibits the visible growth of a microorganism [75]. They are easily determined and reflect several effects such as target interaction, cellular uptake and potential resistance mechanisms of the microorganism. MIC values are therefore widely used, also in studies on muraymycin analogues (e.g., [22,76–78]) and have been the basis of many structure–activity relationship studies (see below).

This bacterial growth assay, however, does not elucidate the inhibitory potency of the potential antimicrobial solely against the target protein MraY. Thus, another assay system that is not based on the interaction with whole cells but only with the target protein is required. For MraY, there are three different assays available that provide such inhibition data: i) a fluorescence-based and ii) a radioactivity-based assay as well as iii) a relatively new Förster resonance energy transfer (FRET)-based method.

The fluorescence-based assay was developed by Bugg et al. [79–80] and uses a fluorescently labelled (dansylated) analogue of the MraY substrate UDP-Mur*N*Ac-pentapeptide. The reaction of this substrate analogue with undecaprenyl phosphate leads to an increase in fluorescence intensity that can be used as a measure for enzymatic activity (e.g., [74,78]). The assay reported by Bouhss et al. [81] uses a radioactively labelled UDP-Mur*N*Ac-pentapeptide and thin layer chromatography (TLC) separation of undecaprenyl-linked Mur*N*Ac-pentapeptide from unreacted substrate (e.g., [77,82]). The third assay was introduced in 2012 by Shapiro et al. and uses a FRET system with the FRET donor attached to the UDP-Mur*N*Ac-pentapeptide and the FRET acceptor in a detergent or detergent/lipid micelle that also hosts the MraY protein [83].

The overexpression and purification of the transmembrane protein MraY is challenging. MraY from different bacterial strains was heterologous overexpressed in *E. coli* and was used in assays mentioned above as a crude cellular membrane preparation or as a detergent-solubilised membrane protein mixture [79,84]. A purification to homogeneity was reported for MraY from *B. subtilis* by Bouhss et al. in 2004 [81] and for the congener from *Aquifex aeolicus* by Lee et al. in 2013 [71]. Wang, Bernhard et al. achieved a cell-free production of MraY from *B. subtilis* and *E. coli*, also experiencing the need of pronounced adjustments in expression conditions [85].

### Synthetic access

**Scheme 1:**
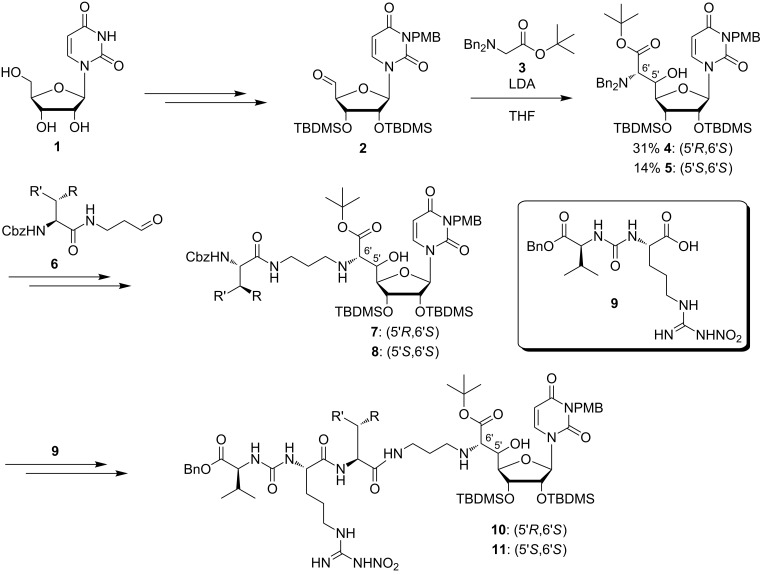
First synthetic access towards simplified muraymycin analogues as reported by Yamashita et al. [76].

Following the isolation of muraymycins [22], a group of scientists from Wyeth reported the semisynthetic access towards 16 derivatives of muraymycin C1 for structure–activity relationship (SAR) studies [86]. At the same time, a first set of fully synthetic structurally simplified muraymycin analogues was described [76]. Starting from uridine (**1**), protected uridine-5'-aldehyde **2** was prepared in four steps (Scheme 1) [87–88]. This was followed by an aldol reaction of aldehyde **2** with *N*,*N*-dibenzylglycine *tert*-butyl ester (**3**) [89] and LDA as a key step of the synthesis (Scheme 1). The resultant products were the two 5'-epimers **4** (5'*R*,6'*S*) in 31% yield and **5** (5'*S*,6'*S*) in 14% yield, which could be separated by column chromatography. After debenzylation, the resultant primary amines were connected with amido aldehydes **6** substituted with different moieties R and R' by reductive amination with R being either a hydroxy group or a hydrogen and R' representing an alkyl, allyl, ester or a protected amino moiety. This led to many truncated muraymycin analogues based on the structures **7** and **8** [76]. Cbz deprotection and subsequent peptide coupling with the L-arginine-L-valine-derived urea dipeptide **9** gave various full-length muraymycin analogues **10** and **11** [76]. Some of the truncated and the full-length compounds were able to inhibit lipid II formation. These active compounds are discussed in the section on structure–activity relationship (SAR) studies.

**Scheme 2:**
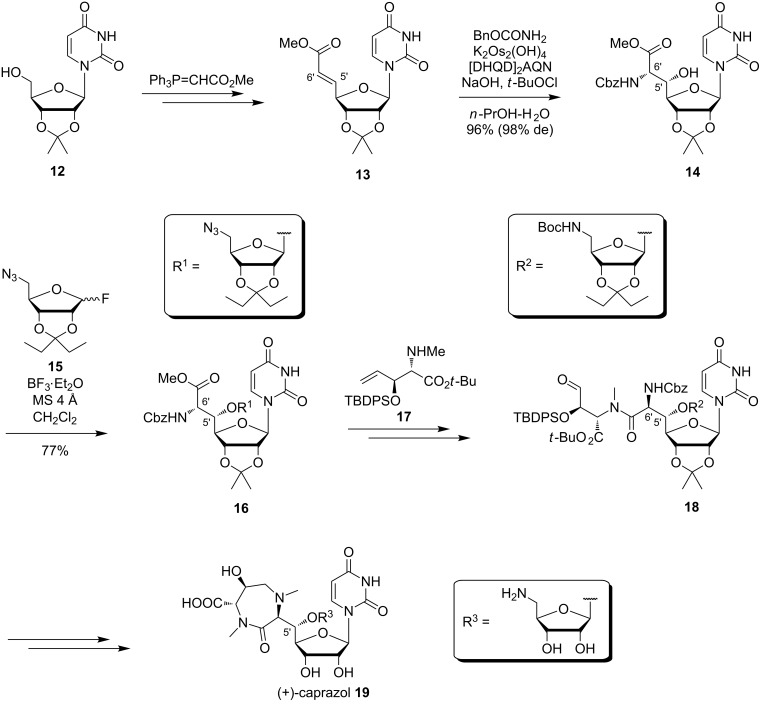
Synthesis of (+)-caprazol (**19**) reported by Ichikawa, Matsuda et al. [92].

In 2005, Ichikawa, Matsuda et al. reported the synthesis of (+)-caprazol [90–92] which contains the same uridine-derived core structure as the muraymycins. The latest and optimised synthesis is shown in Scheme 2 [92]. Oxidation of the isopropylidene-protected uridine **12** to the 5'-aldehyde and a Wittig reaction [93] gave olefin **13**. The key step was a subsequent asymmetric Sharpless aminohydroxylation [94] furnishing (5'*S*,6'*S*)-nucleosyl amino acid **14** in 96% yield (98% de) [92,95]. A novel β-selective glycosylation of the 5'-hydroxy group was also established. Thus, **14** was reacted with the ribosyl fluoride **15** and BF_3_·Et_2_O, which afforded the glycosylated product **16** in 77% yield and with a β/α-selectivity of 24:1 [91–92]. This reaction was followed by an azide reduction, Boc protection, saponification of the ester, peptide coupling with the amino acid **17**, oxidative cleavage of the double bond to give **18** and an intramolecular reductive amination in order to construct the seven-membered ring. Methylation with subsequent acidic global deprotection led to the target compound (+)-caprazol (**19**) [90,92].

**Scheme 3:**
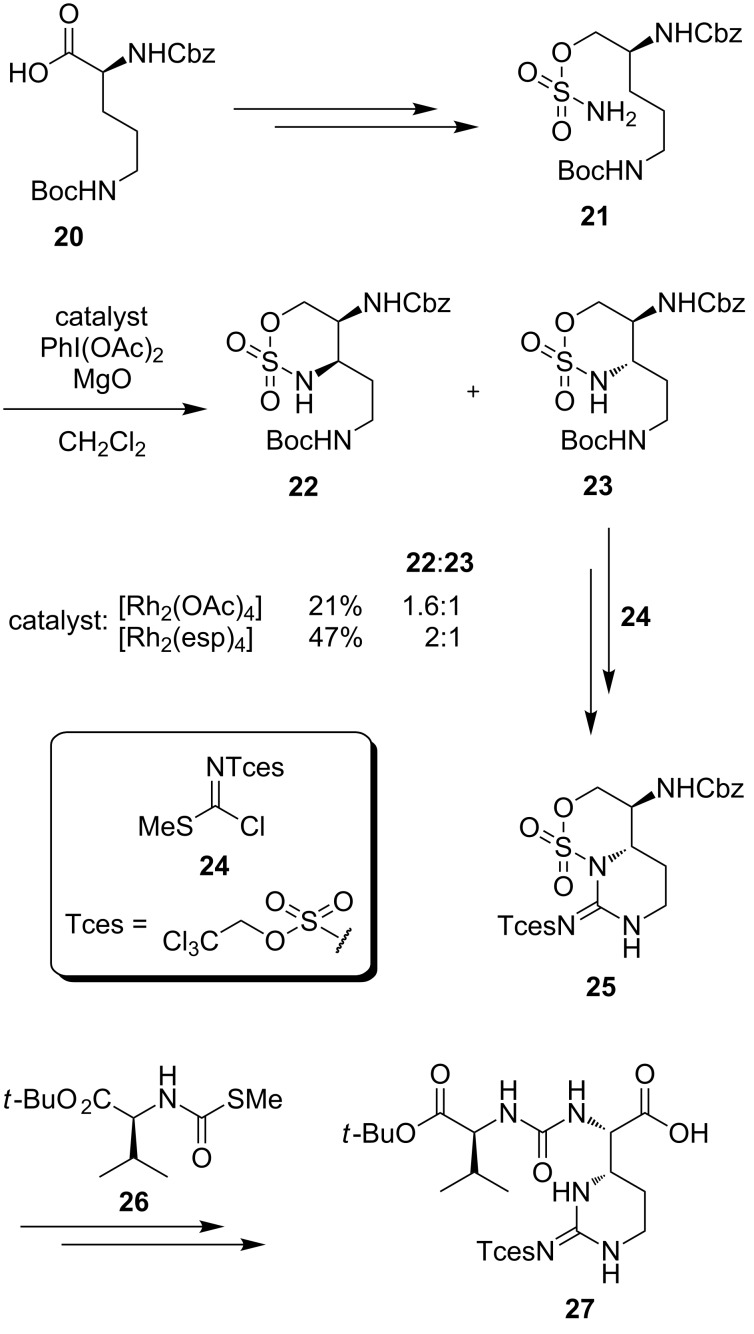
Synthesis of the epicapreomycidine-containing urea dipeptide via C–H activation [96–97].

For the synthesis of muraymycins, Ichikawa, Matsuda et al. furthermore developed a new route towards the epicapreomycidine-containing urea dipeptide unit via C–H activation (Scheme 3) [96–97]. For this purpose, the commercially available δ-*N*-Boc-α-*N*-Cbz-L-ornithine (**20**) was transformed into sulfamate **21**. Subsequently, the C–H insertion representing the key step of this synthesis was examined with two different catalysts and different reaction conditions. Despite different ratios in the outcome of the C–H insertion in favour of the unwanted diastereomer **22**, the synthesis was finished with the desired minor component **23**. Boc deprotection followed by reaction with guanidinylation reagent **24** gave bicyclic compound **25**. The next steps included a desulfonylation and the reaction with **26** leading to protected epicapreomycidine-containing urea dipeptide **27** [96–97].

**Scheme 4:**
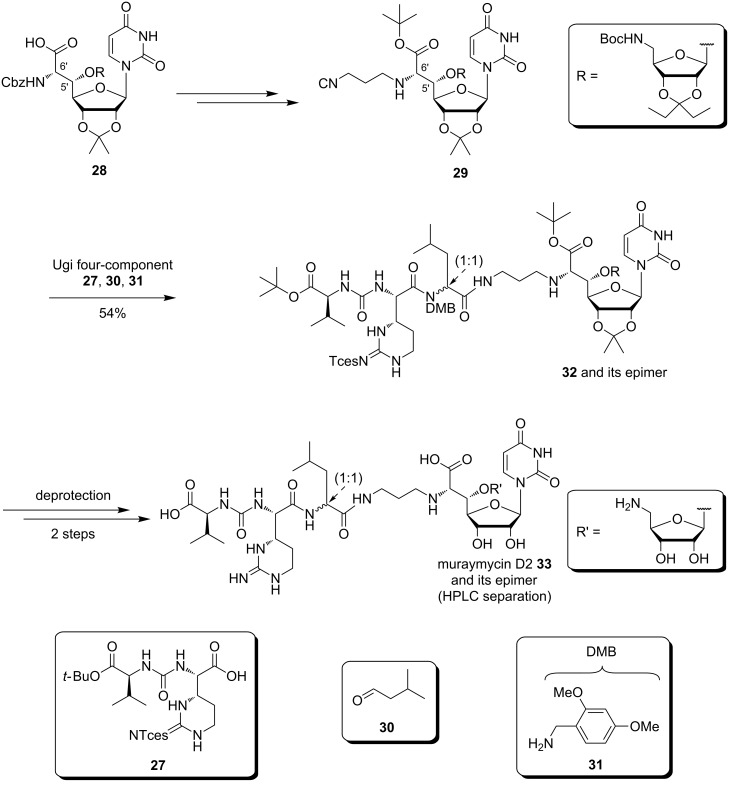
Synthesis of muraymycin D2 and its epimer reported by Ichikawa, Matsuda et al. [96–97].

Starting from the uridine derivative **28** used in the synthesis of (+)-caprazol, Ichikawa and Matsuda built up muraymycin D2 and its epimer (Scheme 4). They used an Ugi four-component reaction with an isonitrile derivative **29** obtained from the uridine-derived core structure **28**, aldehyde **30**, amine **31** and the urea dipeptide building block **27**. A two-step global deprotection then gave the desired muraymycin D2 and its epimer which could be separated by HPLC [96–97].

**Scheme 5:**
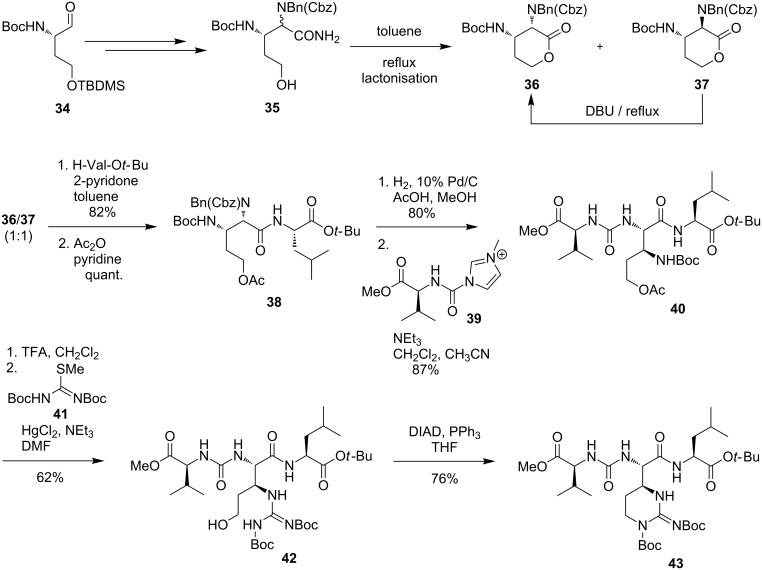
Synthesis of the urea tripeptide unit as a building block for muraymycins reported by Kurosu et al. [98].

In 2012, Kurosu et al. also reported the synthesis of potential key intermediates for the total synthesis of muraymycins (Scheme 5) [98]. A fully protected ureidomuraymycidine tripeptide was prepared through lactone opening followed by urea formation and a final Mitsunobu ring closure as key steps. A Strecker reaction of the benzylimine **34** followed by several steps afforded the alcohol **35**. A thermal lactonisation as a first key step of the synthesis led to a 1:1 mixture of the two epimers **36** and **37**, and the undesired lactone **37** could be epimerised and converted into **36** by treatment with DBU [98]. Epimerisation and simultaneous lactone opening could be achieved in another key step using L-valine *tert*-butyl ester. Acetylation of the thus formed primary alcohol resulted in compound **38**. This was followed by benzyl and Cbz deprotection and the subsequent urea formation with the imidazolium salt **39** to furnish tripeptide **40**. After Boc deprotection, the resultant amine was guanidinylated using isothiourea **41**. The thus obtained precursor **42** was treated with DIAD and PPh_3_ in a final step for an intramolecular Mitsunobu ring closure to finish the synthesis of the fully protected ureidomuraymycidine **43** (Scheme 5) [98].

**Scheme 6:**
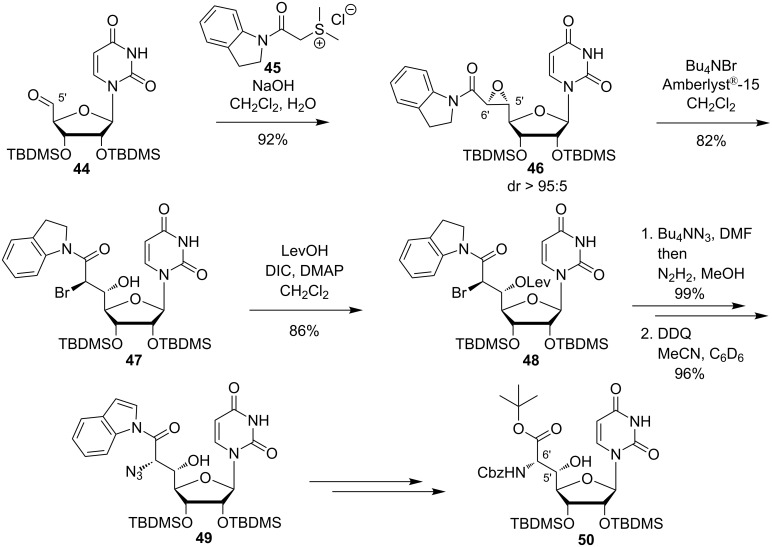
Synthesis of the uridine-derived core structure of naturally occuring muraymycins reported by Ducho et al. [78,99].

In 2010, Ducho et al. reported an alternative synthesis of the naturally occurring uridine-derived muraymycin core structure (Scheme 6) [78,99]. The key step of their route was a sulfur-ylide reaction with high substrate-controlled diastereoselectivity [100–102]. This epoxide-forming sulfur-ylide reaction had been established before by Sarabia et al. [103–104]. After some initial confusion regarding the stereochemical configuration of the epoxide product, it could be unambiguously proven that the transformation of uridine-5'-aldehyde **44** with sulfonium salt **45** under basic conditions furnished epoxide **46** with high diastereoselectivity (Scheme 6). Subsequent ring opening of this epoxide with Bu_4_NBr resulted in bromohydrine **47**, followed by levulinyl (Lev) protection of the hydroxy group (product **48**). Nucleophilic substitution at the 6'-position with Bu_4_NN_3_ gave the naturally occurring (5'*S*,6'*S*)-stereochemistry of the uridine core structure in a double inversion manner [78,99]. DDQ oxidation then provided indolamide **49**. Hydrolysis of the amide, formation of the synthetically more versatile *tert*-butyl ester, azide reduction and final Cbz protection resulted in the uridine-derived building block **50** for the synthesis of naturally occurring muraymycins (Scheme 6). Furthermore, 5'- and 6'-*epi* analogues of muraymycins were also synthesised via suitable epoxide precursors by Ducho et al. [105].

**Scheme 7:**
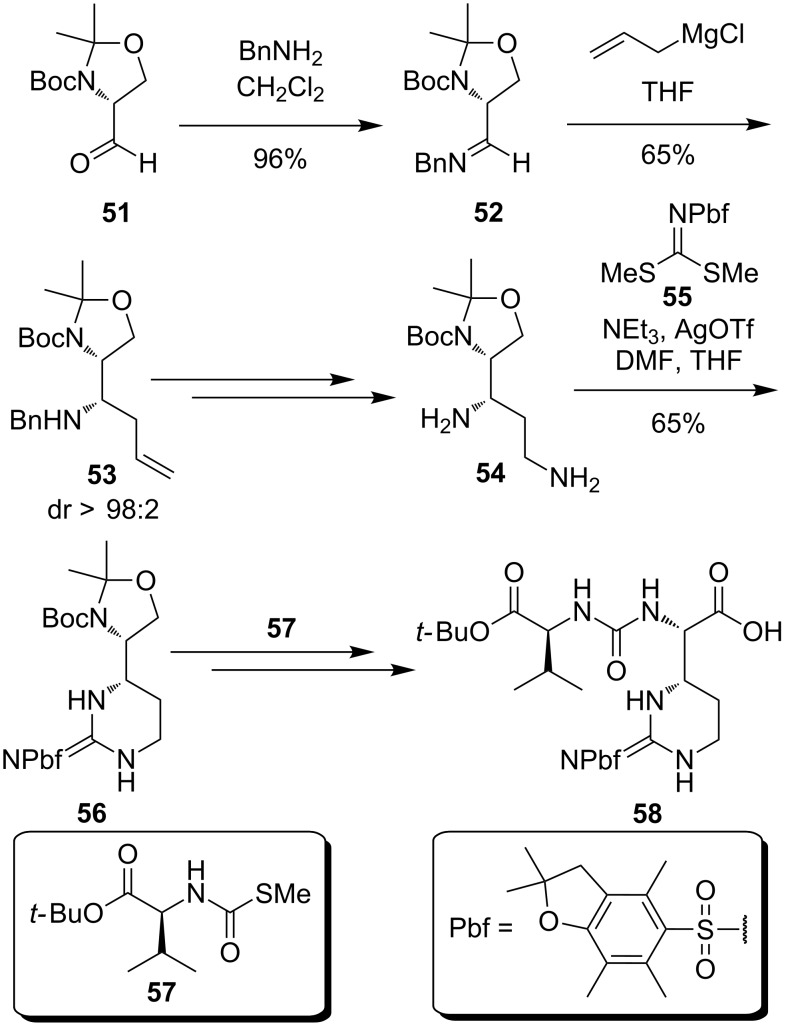
Synthesis of the epicapreomycidine-containing urea dipeptide from Garner's aldehyde reported by Ducho et al. [78].

Ducho's synthesis of epicapreomycidine (Scheme 7) started from the (*R*)-configured Boc-protected Garner aldehyde **51** [106], which was transformed into the *N*-benzylimine **52**. The latter was then diastereoselectively converted with a Grignard reagent into the amine **53** as a key step of the synthesis [78]. Cbz protection followed by ozonolysis with subsequent reductive amination and hydrogenolysis led to the 1,3-diamine **54**. The cyclisation to the guanidine functionality was achieved with the novel guanidinylation reagent **55**. With the protected epicapreomycidine precursor **56** in hand, the Boc and acetonide protecting groups were removed. Urea formation with the valine derivative **57** with final oxidation of the primary hydroxy function afforded the desired dipeptide **58** [78] (Scheme 7).

**Scheme 8:**
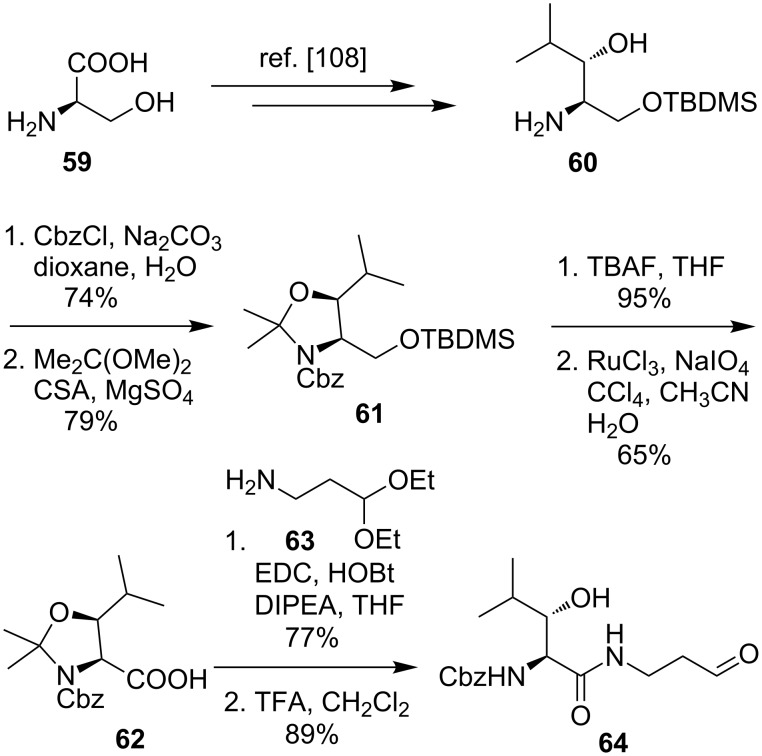
Synthesis of a hydroxyleucine-derived aldehyde building block reported by Ducho et al. [107].

Furthermore, Ducho et al. synthesised the hydroxyleucine moiety found in naturally occurring muraymycins of classes A to C (Scheme 8) [107]. Adapting a strategy developed by Zhu et al., D-serine (**59**) was stereoselectively converted into the protected amino alcohol **60** [108]. Key intermediate **60** was then Cbz- and acetonide protected to give **61**. A sequence of desilylation and oxidation furnished the acid **62**. Peptide coupling with amine **63** and acidic deprotection then afforded the desired aldehyde **64**, which already contained the muraymycin linker unit (Scheme 8) [107]. Together with the uridine core structure **50** and the urea dipeptide **58**, the aldehyde **64** was the third building block of Ducho's envisioned stereocontrolled tripartite route towards muraymycins, in contrast to Ichikawa's and Matsuda's modular multicomponent, but non-stereocontrolled approach (see above).

**Scheme 9:**
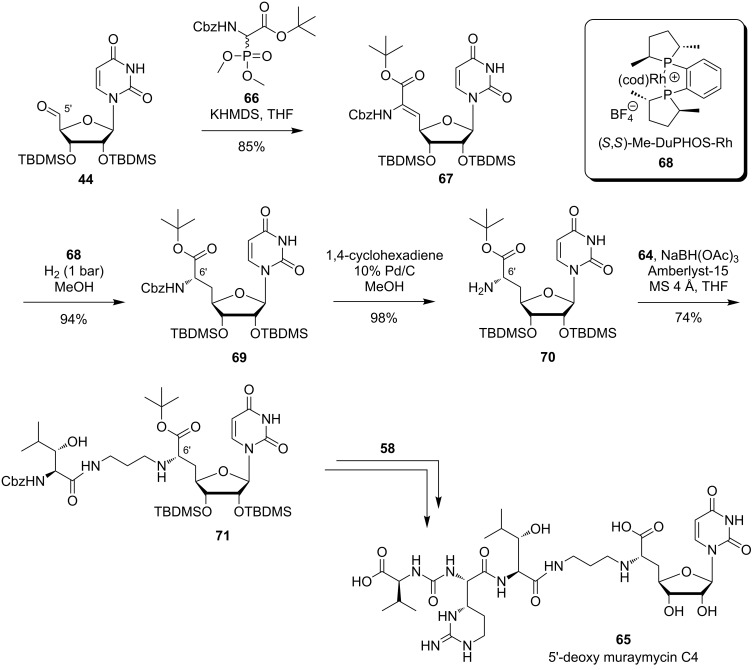
Synthesis of 5'-deoxy muraymycin C4 (**65**) as a closely related natural product analogue [78,109–110].

This novel tripartite approach was then used by Ducho et al. to synthesise the structurally simplified natural product analogue 5'-deoxy muraymycin C4 (**65**), which formally differs from the parent natural product only by absence of one oxygen atom (Scheme 9) [78,109–110]. Starting from protected uridine-5'-aldehyde **44**, the first key step of the synthesis was a (*Z*)-selective Wittig–Horner reaction with phosphonate **66** [111] in order to obtain the didehydro amino acid **67**. The next important step of this route was an asymmetric catalytic hydrogenation [112–113] with the chiral Rh(I)–DuPHOS catalyst **68** to prepare the (6'*S*)-configured product **69** [109–110]. Subsequent hydrogenolytic cleavage of the Cbz group gave the nucleosyl amino acid **70**. To complete the tripartite approach, the reductive amination with the aldehyde **64** furnished **71**, and Cbz deprotection and peptide coupling with the epicapreomycidine-containing urea dipeptide **58**, followed by acidic global deprotection, gave the desired 5'-deoxy muraymycin C4 (**65**) (Scheme 9) [78].

In addition to the described synthetic routes, a range of other muraymycin analogues has been prepared. In the interest of conciseness, this synthetic work is not discussed here, but the biological properties of such analogues will be summarised in the following section on SAR studies.

### Structure–activity relationship studies

With various structurally diverse compounds at hand, the stage has been set for SAR studies on muraymycins. The antimicrobial activities found by McDonald et al. introduced muraymycins as a promising subject of study [22]. The naturally occurring muraymycins isolated from *Streptomyces* guided first insights into the structural features essential for MraY inhibition. For the most active member of the family, i.e., muraymycin A1, antibiotic activity could be found against various bacteria ranging from *Staphylococci* with MIC values of 2 to 16 μg/mL, *Enterococci* with 16 μg/mL and higher to some Gram-negative bacteria (8 μg/mL). Against an *E. coli* mutant with increased membrane permeability, an MIC value below 0.03 μg/mL was obtained, suggesting that inhibition is a matter of cellular uptake of the compound. In vivo efficacy was demonstrated for muraymycin A1 with an ED_50_ of 1.1 mg/kg in *Staphylococcus aureus*-infected mice.

Five of the 19 naturally occurring compounds (i.e., muraymycins A1, A5, B6, C2 and C3) were capable of inhibiting both MraY and peptidoglycan synthesis at the lowest concentration tested (IC_50_ = 0.027 μg/mL), which represented activities comparable to those of liposidomycin C (0.05 μg/mL) and mureidomycin A (0.03 μg/mL). As a general trend, higher antimicrobial activities were found for acylated compounds, in particular with longer and functionalised fatty acid side chains.

**Figure 7:**
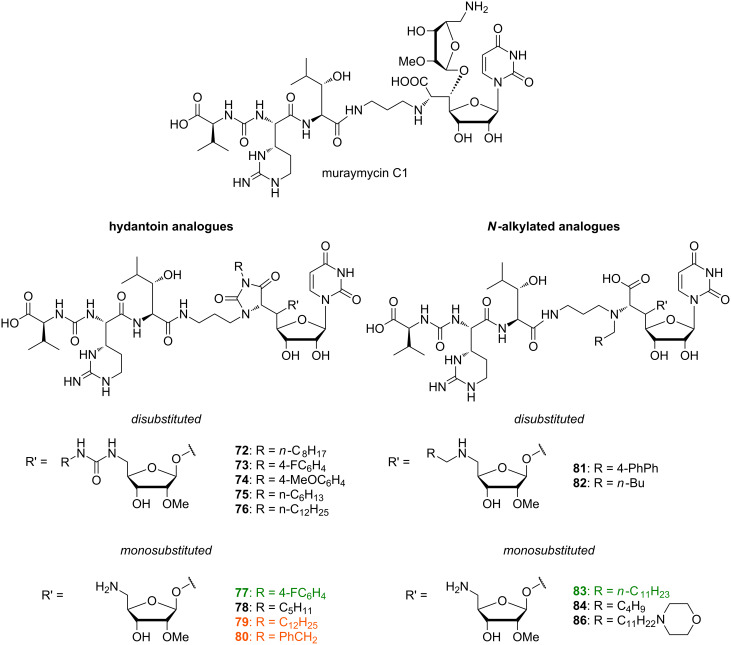
Summary of modifications on semisynthetic muraymycin analogues tested by Lin et al. [86]. Most active compounds are highlighted (in vitro inhibition of lipid II formation at 6.25 μg/mL: orange; 25–50 μg/mL: green).

Lin et al. employed a semisynthetic approach for modifications of muraymycin C1 as starting point of their SAR studies (Figure 7) [86]. In accordance with the results reported by McDonald et al., their work was based on the assumption that the cellular uptake required for MraY inhibition is mainly dependent on fatty acids connected to the hydroxyleucine moiety. The attachment of lipophilic groups on either the primary or both the primary and secondary amino function was supposed to have similar effects. The muraymycin derivatives **72**–**86** were thus evaluated against the target in a coupled MraY–MurG in vitro assay employing radiolabelled UDP-*N*-acetylglucosamine. Disubstituted analogues were not active at the concentrations tested, suggesting that one free amino group is vital for activity. Hydantoin-derived compounds **79** with C_12_H_25_ and **80** with PhCH_2_ as residues R at the hydantoin moiety gave the best results with inhibition of lipid II formation at 6.25 μg/mL, which is comparable to muraymycin C1. Good activity was also found for hydantoin derivative **77** with the 4-FC_6_H_4_ substituent, showing inhibition of lipid II formation at 25 μg/mL. The only *N*-alkylated derivative inhibiting in the same order of magnitude was **83** with *n*-C_11_H_23_ substitution. However, activities of the other compounds within this group also coincided with the previous observation that lipophilic compounds were more active. Overall, the tested monosubstituted hydantoin derivatives confirmed the assumed correlation between inhibitory activities and lipophilicity of the substituent.

**Figure 8:**
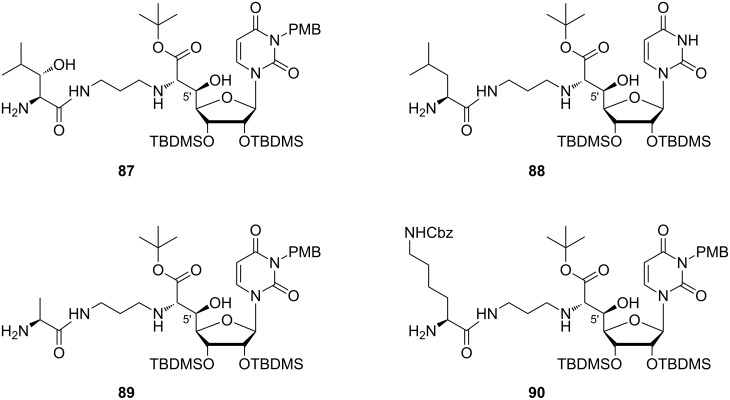
Bioactive muraymycin analogues identified by Yamashita et al. [76]*.*

Yamashita et al. studied truncated muraymycin analogues lacking the lipophilic side chain as described in the section on synthetic access (compounds of type **7**, **8** and **10**) [76]. The activities measured in a soluble peptidoglycan assay indicated a stereochemical preference for the (5'*S*)-configuration, contrary to the results of MIC value determination. Further studies were then carried out with (5'*R*)-derivatives only, i.e., with 5'-epimers of the parent natural products. The influence of protecting groups was examined applying a strategy of stepwise deprotection. This led to the observation that fully protected compounds were not active at all, as well as the completely deprotected analogues. Remarkably, some partially protected congeners **87**–**90** with the free terminal amino group were found to show good inhibition (MIC = 1–16 μg/mL) of the growth of Gram-positive bacteria including *S. aureus* and *E. faecalis* strains, with best results obtained for **88** (Figure 8). Evaluation of the inhibition of lipid II formation revealed the importance of the substitution pattern of the terminal amino acid.

In 2010 and 2011, Ichikawa, Matsuda et al. published SAR studies with a range of synthetic muraymycin analogues [77,114]. The IC_50_ values were measured in an in vitro assay mentioned above to examine the inhibitory activity of the prepared analogues against the target enzyme. MIC values were determined against several bacterial strains. The inhibitory activities of the synthesised muraymycin D2 **33** (with an L-leucine unit) and its epimer (with a D-leucine unit) on the purified MraY enzyme from *B. subtilis* were determined. Both compounds showed good inhibitory activities with IC_50_ values of 0.01 μM and 0.09 μM, respectively. However, their antibacterial activities against several Gram-positive bacteria (*S. aureus, E. faecalis, E. faecium*) were low (MIC values up to 64 μg/mL). In comparison to the analogues of the A and B series, which showed good antibacterial activities (see above), muraymycin D2 (**33**) and its epimer lack the hydrophobic side chain at the leucine moiety [22]. It was postulated that this lipophilic side chain may not be necessary for target inhibition, but for cellular uptake through the lipid bilayer of the cytoplasmic membrane, as an increased lipophilicity is advantageous for this [77,114].

**Figure 9:**
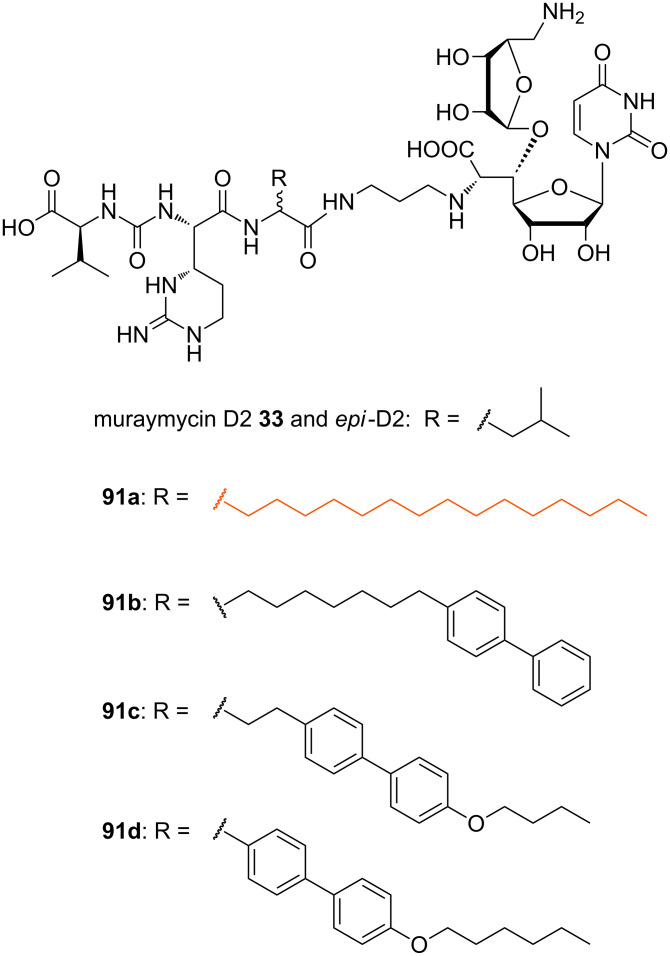
Muraymycin D2 and several non-natural lipidated analogues **91a**–**d** [77,114].

Consequently, several lipophilic derivatives **91a**–**d** were prepared (Figure 9). Long-chain lipophilic amino acids were incorporated into the muraymycin core structure as a simplified replacement of the *O*-acylated hydroxyleucine moiety. Compound **91a** (highlighted in orange) with the pentadecyl side chain showed the best activity as an MraY inhibitor (IC_50_ = 0.33 μM (with L-leucine moiety), IC_50_ = 0.74 μM (with D-leucine moiety)), but relative to muraymycin D2 and its epimer, this implied a 33-fold and 8-fold, respectively, decrease of inhibitory activity. In bacterial growth assays, the analogue **91a** exhibited the best MIC values ranging between 0.25 μg/mL and 4 μg/mL (see Table 2). These values were comparable to those of the naturally occurring congeners of the A and B series [22]. Generally, derivatives with the naturally occurring L-configuration in the leucine moiety showed slightly better activities. These lipophilic analogues were also tested for cytotoxicity towards Hep G2 cells and showed no cytotoxicity (IC_50_ > 100 μg/mL) [114].

**Table 2:** Inhibitory (against MraY) and antibacterial activities of non-natural lipophilic muraymycin analogues [77,114].

Compound	(L-Leu) (D-Leu)	IC_50_ (μM)^a^	MIC (μg/mL)^b^

muraymycin D2 (**33**)		0.01 0.09	>64 >64
**91a**		0.33 0.74	2–4 0.25–4
**91b**		n.d.	4–16 4–16
**91c**		n.d.	16–64 4–64
**91d**		n.d.	4–8 4–16
**92a**		n.d.	2–4 2–4
**92b**		n.d.	1–2 2–4
**92c**		n.d.	2–8 4–8
**92d**		n.d.	4–8 4–8
**92e**		n.d.	4–8 4–8

Compound (L-Leu)		IC_50_ (μM)^a^	MIC (μg/mL)^b^

**92f**		n.d.	4–8
**92g**		n.d.	4
**92h**		n.d.	4–8
**93**		5	32 to ≥64

^a^Inhibitory activities were determined against purified MraY enzyme from *B. subtilis* [77]; ^b^MIC values were determined for different strains of *S. aureus*, *E. faecalis* and *E. facium* including some multiresistant strains [77]; n.d. = not determined.

**Figure 10:**
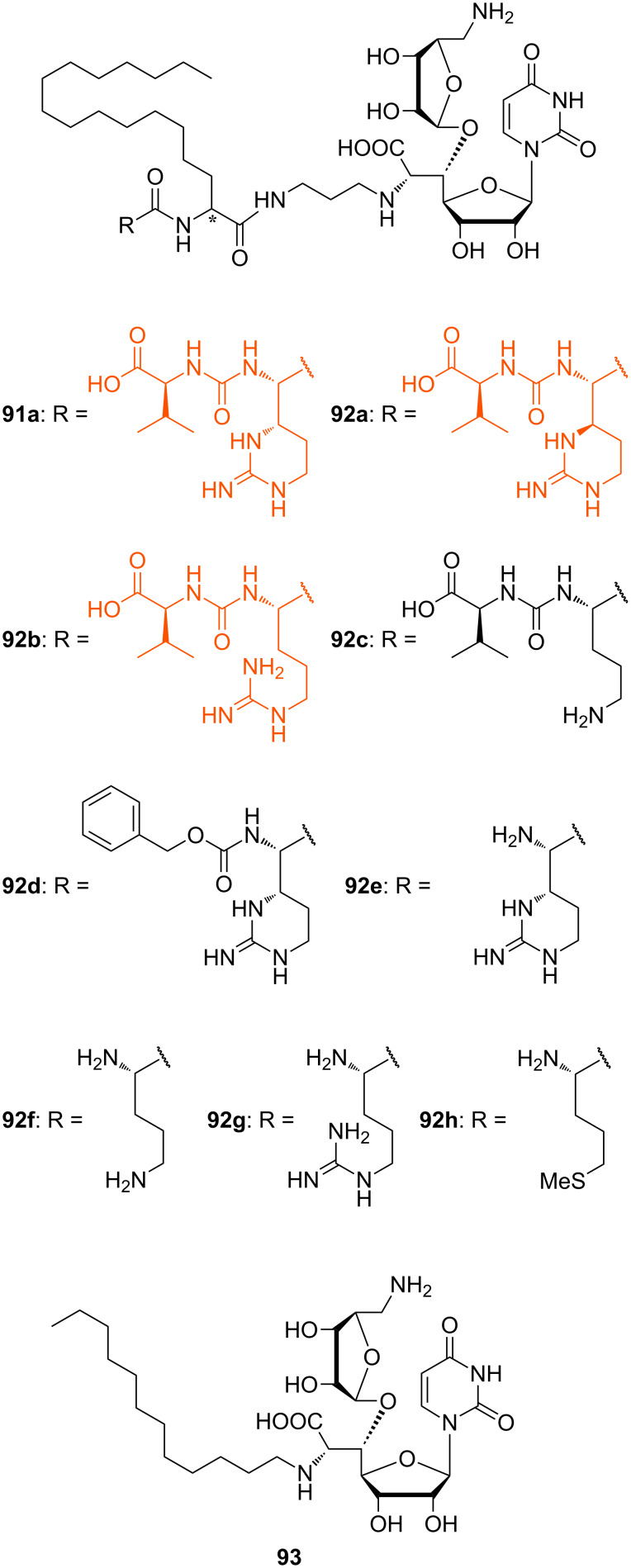
Non-natural muraymycin analogues with varying peptide structures [77,114].

In another series of analogues with different peptide units, the pentadecyl side chain of **91a** was kept. The L-epicapreomycidine (L-*epi*-Cpm) unit of **91a** was replaced by L-capreomycidine (L-Cpm, **92a**), L-arginine (L-Arg, **92b**) and L-ornithine (L-Orn, **92c**) in order to investigate the role of the cyclic guanidine functionality (Figure 10) [77].

**Figure 11:**
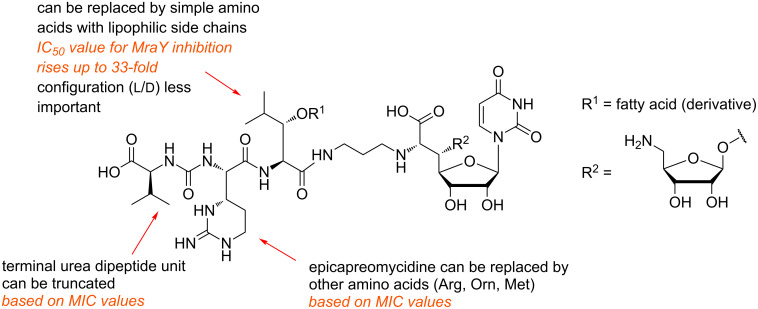
SAR results for several structural variations of the muraymycin scaffold.

These compounds were all active against MRSA and VRE with varying MIC values (Table 2). The most active analogues of this series were **92a** and **92b** (Figure 10, highlighted in orange) with MIC values between 1 μg/mL and 4 μg/mL. Derivatives with unnatural D-stereochemistry in the pentadecyl glycine motif possessed a similar antibacterial activity (potency within factor 2). Truncated analogues lacking the L-valine urea terminus (Cbz-protected **92d** and N-terminally unprotected **92e**) showed only a minor loss of activity (MIC = 4–8 μg/mL) (Table 2). These results indicated that the guanidine motif of analogues **91a**, **92a** and **92b** (MICs between 0.25 μg/mL and 4 μg/mL) is preferred, but that amino analogues **92c** and **92f** still show good activity (MICs between 2 μg/mL to 8 μg/mL). The different stereochemistry at the central leucine unit and the terminal truncation had no crucial effects on the antibacterial activity (Table 2). Truncated derivatives **92f**–**h** (Figure 10) without the L-valine urea terminus contained L-ornithine (L-Orn, **92f**), L-arginine (L-Arg, **92g**) and L-methionine (L-Met, **92h**), respectively. They were also tested and showed reasonable activity against some bacterial strains (MIC = 4–8 μg/mL), which further indicated that significant variations in the peptide moiety are tolerated. The truncated analogue **93** (Figure 10) only consisted of the *N*-alkylated nucleoside core structure. Its inhibitory activity was 6 to 12-fold reduced (IC_50_ = 5 μM) and the antibacterial activity decreased with MIC values between 32 μg/mL and 64 μg/mL. In summary, these systematic SAR studies demonstrated the importance of the lipophilic side chain for the antibacterial activity. The urea dipeptide motif is important for antibacterial activity as well, but it could be diversified with simpler amino acids as well as being truncated in order to provide bioactive analogues. A graphical summary of these results is provided in Figure 11.

**Figure 12:**
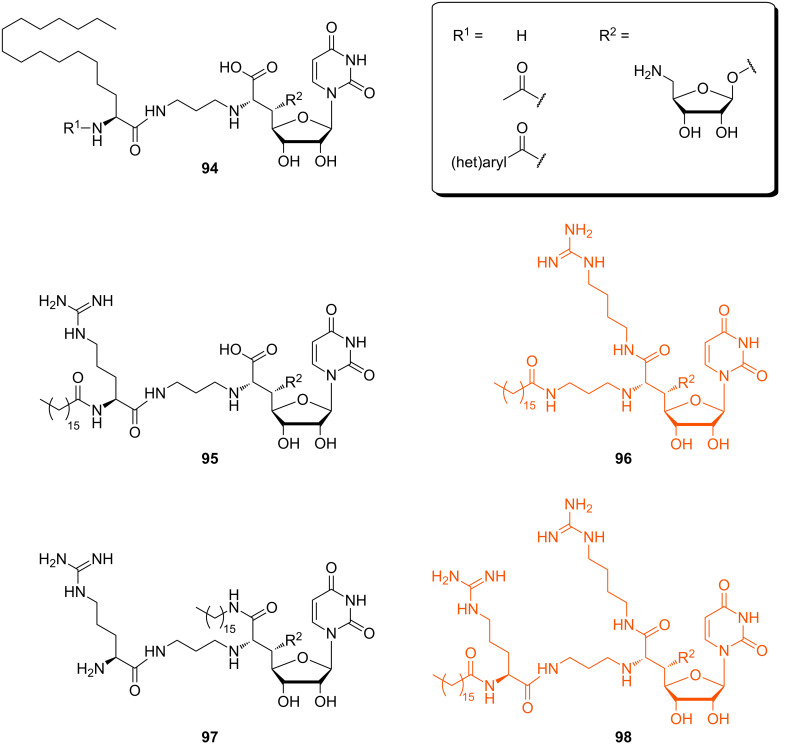
Muraymycin analogues designed for potential anti-*Pseudomonas* activity (most active analogues are highlighted in orange) [115].

In 2014, Ichikawa, Matsuda et al. continued their SAR studies with respect to urgently needed anti-*Pseudomonas* agents [115]. These Gram-negative bacteria possess an outer membrane which acts as an additional permeability barrier, making them generally less sensitive to antibacterial agents. In this context, the aforementioned muraymycin analogues (**91a**, **92a**–**h**) were tested for MraY inhibitory activity again, with MraY enzyme from *S. aureus* (Table 3). However, antibacterial activities against several *Pseudomonas* strains were moderate to low with MICs between 8 μg/mL and >64 μg/mL. Analogue **92g** was the most active congener in this series with MIC values between 8 μg/mL and 32 μg/mL. Compounds **92e** and **92f** showed nearly no activity (MIC = 32 to >64 μg/mL). More lipophilic truncated analogues **94** without the urea dipeptide unit (Figure 12) were synthesised and tested, but they all showed nearly no activity.

**Table 3:** Inhibitory (against MraY) and antibacterial activities of non-natural muraymycin analogues against *Pseudomonas aeruginosa* [115].

Compound	IC_50_ (nM)^a^	MIC (μg/mL)^b^

**91a**, **92a**–**d**	0.7–4.2	≥ 64
**92e**,**f**	2.4–3.8	32 to ≥ 64
**92g**	2.2	8–32
**92h**	8.5	16 to ≥ 64
**94** (R^1^ = -H or -COCH_3_)	2.6–2.7	32 to ≥ 64
**94** (R^1^ = -CO(het)aryl )	6.4–105	≥ 64
**95**	1.6	8–32
**96**	0.14	8–16
**97**	12.2	16–32
**98**	0.60	4–8

^a^Inhibitory activities were determined against MraY enzyme from *S. aureus* [115]; ^b^MIC values were determined for several *P. aeruginosa* strains [115].

These results indicated the importance of the presence of a guanidine residue and a lipophilic side chain for potential antibacterial activity against *Pseudomonas* strains. Hence, several derivatives were prepared in which the positions and numbers of the guanidine groups and the lipophilic side chains were varied in order to optimise their relative orientation for best biological activity. This strategy resulted in the bioactive analogues **95**–**98** (Figure 12). Analogue **95** with an interconversion of the lipid side chain and the guanidine group had a slightly reduced activity compared to lipidated analogue **92g**. Analogue **96** showed an increased antibacterial activity towards some of the tested *Pseudomonas* strains. Analogue **97** is an interconverted version of **96** and displayed a comparatively poor activity. The most active analogue was compound **98** which is a hybrid type of the aforementioned analogues **95**–**97**. The results indicate that a lipophilic side chain and guanidine groups are necessary for antibacterial potency. Compounds **95**–**98** showed antibacterial activity, with the branched-type compound **96** (MIC values between 8 μg/mL and 16 μg/mL) and the hybrid-type compound **98** (MIC between 4 μg/mL and 8 μg/mL) being the most active congeners. A limitation of both analogues **96** and **98** is their increased cytotoxicity against HepG2 cells with IC_50_ values of 4.5 μg/mL and 34 μg/mL, respectively. Further, the metabolic stability was studied in vitro for the analogues **95**, **96** and **98** using human or rat liver microsomes and all of them proved to be reasonably stable [115].

In 2014, Ducho et al. reported the synthesis of 5'-deoxy muraymycin C4 (**65**, see above) [78]. Biological assays revealed that **65** inhibited the MraY enzymes of *E. coli* and *S. aureus* with potencies in the range of tunicamycins. The antibacterial activity of **65** was tested against some selected *E. coli* and *S. aureus* strains although the lack of a lipophilic moiety indicated that the compound should not be a potent antibiotic. However, an unexpected moderate activity against *E. coli* DH5 alpha was observed, whereas **65** was weakly active against *E. coli* strain ΔtolC but not active against the *S. aureus* Newman strain. Further studies indicated excellent plasma and metabolic stability and no cytotoxicity. Overall, the structurally simplified 5'-deoxy muraymycin scaffold **65** may therefore be useful for further antibacterial development. It should also be noticed that it has inspired the design of a novel oligonucleotide backbone modification [116–117].

### Biosynthesis

**Scheme 10:**
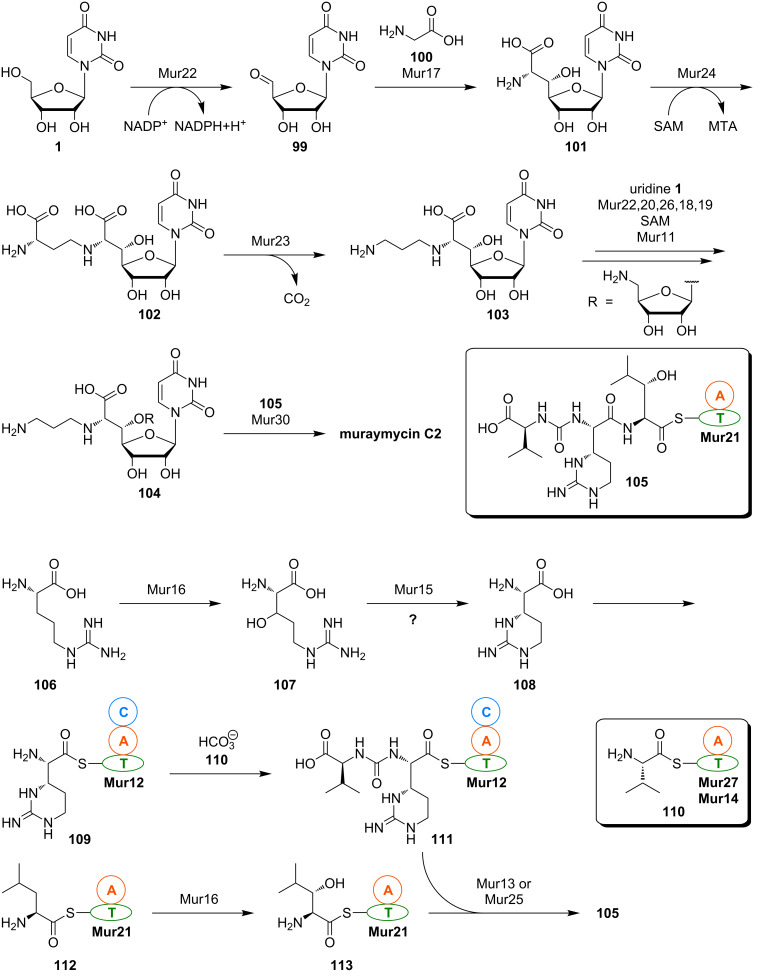
Proposed outline pathway for muraymycin biosynthesis based on the analysis of the biosynthetic gene cluster by Chen, Deng et al. [118]. MTA = 5'-deoxy-5'-(methylthio)adenosine.

So far, there are only limited insights into muraymycin biosynthesis. The biosynthetic gene cluster for the formation of muraymycins in *Streptomyces* sp. NRRL 30471 has been identified by Chen, Deng et al. in 2011 [118]. The sequence analysis revealed the cluster to contain 33 open reading frames (ORFs) with 26 of them being involved in muraymycin formation. Based on their elucidation of the gene cluster and sequence homologies, Chen, Deng et al. proposed an outline pathway for muraymycin biosynthesis (Scheme 10).

According to this biosynthetic proposal, uridine (**1**) is enzymatically oxidised to give uridine-5'-aldehyde **99**. Aldehyde **99** then supposedly undergoes an aldol addition with glycine **100** as the enol(ate) component, thus furnishing the amino acid–nucleoside hybrid 5'-*C*-glycyluridine (GlyU, **101**). Alkylation of the 6'-amino group is then achieved by reaction with *S*-adenosyl methionine (SAM), and the resultant intermediate **102** is decarboxylated to provide diamine **103**. Attachment of the aminoribosyl moiety (which is supposedly also derived from uridine (**1**) over several enzymatic steps) finally affords the aminopropyl-substituted 5'-*O*-aminoribosylated GlyU core structure **104**. Transformation of **104** with the thioester-activated peptide moiety **105** then gives muraymycin C2 (Scheme 10), which is speculated to serve as an intermediate en route to other muraymycins, in particular towards *O*-lipidated congeners of the A and B series (see Figure 2).

A fragmented non-ribosomal peptide synthetase (NRPS) system appears to be responsible for the assembly of the urea tripeptide building block **105**. However, the non-proteinogenic amino acids need to be formed first. It has been proposed that L-arginine (**106**) undergoes 3-hydroxylation (giving 3-hydroxy-L-arginine (**107**)) and subsequent ring closure to furnish L-epicapreomycidine ((2*S*,3*S*)-capreomycidine, **108**), that is then activated as thioester **109** (Scheme 10). This proposal is based on the elucidated formation of the epimeric amino acid L-capreomycidine ((2*S*,3*R*)-capreomycidine) as part of viomycin biosynthesis in *Streptomyces vinaceus*. In this producing organism, L-arginine is diastereoselectively hydroxylated to afford (3*S*)-3-hydroxy-L-arginine. The ring-closure reaction then occurs with formal inversion of the β-stereocenter (but quite likely through an aza-Michael addition to the α,β-unsaturated intermediate) [119–121]. The exact stereochemical course of epicapreomycidine formation in muraymycin biosynthesis is unclear though as the stereochemical configuration at C-3 of the intermediate 3-hydroxy-L-arginine (**107**) has not been identified yet. It cannot be ruled out that an epimerisation reaction might be involved in the biosynthesis of **108**, in particular with respect to other epimerisation steps in bacterial biosynthetic pathways [122]. Consequently, synthetic routes towards both 3-epimers of 3-hydroxy-L-arginine have been developed which would also enable the preparation of isotopically labelled congeners for biosynthetic studies [123–124]. It should also be noted that a biomimetic domino guanidinylation–aza-Michael-addition reaction for the synthesis of the capreomycidine scaffold has been developed, which only furnished the target structures as stereoisomeric mixtures though [125].

The epicapreomycidine-derived thioester **109** is proposed to be converted into the urea dipeptide motif with valine derivative **110** and possibly hydrogen carbonate as a C_1_-building block for urea formation, thus furnishing **111**. The 3-hydroxy-L-leucine moiety might be obtained by stereoselective enzymatic β-hydroxylation of thioester-activated L-leucine **112**, which leads to the formation of **113**. Finally, peptide formation by condensation of **111** with **113** affords the complete thioester-activated urea tripeptide unit **105** (Scheme 10). One interesting aspect of this biosynthetic proposal by Chen, Deng et al. is that they assume the putative dioxygenase Mur16 to catalyse β-hydroxylations of two structurally distinct amino acid substrates, i.e., L-arginine (**106**) and thioester-activated L-leucine **112**.

**Scheme 11:**
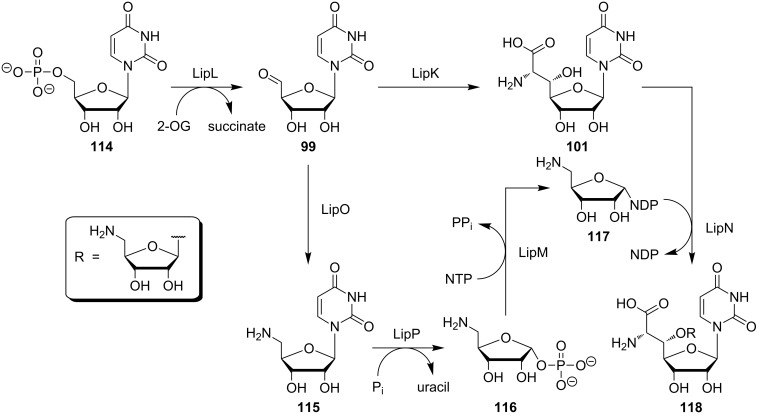
Biosynthesis of the nucleoside core structure of A-90289 antibiotics (which is identical to the muraymycin nucleoside core) according to the studies of Van Lanen et al. [126]. 2-OG = 2-oxoglutarate.

As pointed out, there is a lack of experimental insights into muraymycin biosynthesis beyond the elucidation of its gene cluster. However, Van Lanen et al. have studied the early steps of the biosynthesis of A-90289 nucleoside antibiotics in detail (Scheme 11) [126]. The A-90289 subclass is structurally closely related to caprazamycins and liposidomycins, and its aminoribosylated nucleoside core is identical to that of muraymycins (Figure 2). This supports the assumption that the early steps of the biosynthesis of all these subclasses are probably highly similar, if not identical. For the A-90289 nucleoside antibiotics, Van Lanen et al. have demonstrated that uridine monophosphate (UMP, **114**) is the actual source of uridine-5'-aldehyde **99**, which is furnished in an oxidative transformation of UMP **114** with the 2-oxoglutarate (2-OG)-dependent non-haem Fe(II)-oxygenase LipL [127]. This result challenges the proposal by Chen, Deng et al. that aldehyde **99** might be formed by oxidation of uridine (**1**) in muraymycin biosynthesis. Aldehyde **99** then undergoes the aforementioned aldol-type transformation to GlyU **101**, catalysed by the enzyme LipK. However, aldehyde **99** also serves as a source of the aminoribosyl moiety. Thus, it is converted into 5'-amino-5'-deoxyuridine (**115**) in a transamination reaction mediated by LipO. This is followed by the LipP-catalysed displacement of the uracil with a phosphate moiety to afford 5-amino-5-deoxyribose-1-phosphate (**116**). The LipM-mediated reaction of ribosyl phosphate **116** with a nucleoside triphosphate (NTP) then yields nucleoside diphosphate (NDP)-aminoribose **117**. Finally, aminoribosylation of **101** with glycosyl donor **117**, catalysed by glycosyltransferase LipN, furnishes the complete nucleoside core structure **118** (Scheme 11). The order of 6'-*N*-(3-aminopropyl) attachment and 5'-*O*-aminoribosylation is not fully clear yet, i.e., it is not elucidated if **101** or 6'-*N*-aminoalkyl intermediate **103** (see Scheme 10) act as the glycosyl acceptor in the aminoribosylation step.

**Scheme 12:**
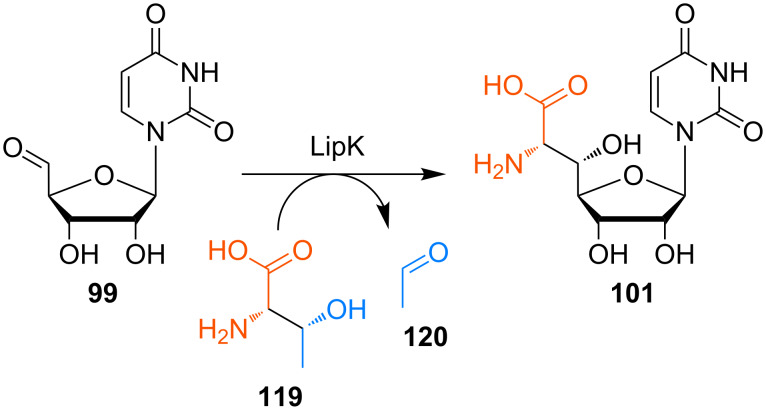
Transaldolase-catalysed formation of the key intermediate GlyU **101** in the biosynthesis of muraymycin-related A-90289 antibiotics [128].

Van Lanen et al. then studied the LipK-catalysed aldol-type formation of GlyU **101** in more detail [128]. Surprisingly and in contrast to Chen's and Deng's proposal, L-threonine (**119**) turned out to be the source of the enol(ate) component instead of glycine (**100**). Hence, LipK was revealed to be a transaldolase mediating a retro-aldol reaction of L-threonine (**119**) towards the enol(ate) and acetaldehyde (**120**), followed by a stereoselective aldol addition of the former to uridine-5'-aldehyde **99** (Scheme 12). Using synthetic reference compounds, it could be proven that (5'*S*,6'*S*)-GlyU **101** is the stereoisomer furnished in this reaction, so that no epimerisation at a later stage of the biosynthetic route is required for the formation of the A-90289 nucleoside antibiotics.

Based on the elucidation of the LipK-mediated reaction, Van Lanen et al. then performed a PCR-based screening of a collection of ≈2500 actinomycete strains for similar transaldolase-encoding genes [129]. They could identify the gene *sphJ* from a *Sphaerisporangium* sp., which encoded the transaldolase SphJ having 51% amino acid sequence identity with LipK. Following detailed characterisation of this enzyme, the *sphJ* gene was employed as a probe to clone the entire genetic locus consisting of 34 putative ORFs. The expression of three selected genes (including *sphJ*) was monitored under different growth conditions. Under the thereby identified optimal conditions, the actinomycete produced a set of four unprecedented MraY-inhibiting nucleoside antibiotics named sphaerimicin A to D [129]. Hence, detailed studies on LipK-like transaldolases led to the discovery of novel antimicrobially active secondary metabolites.

It remains to be proven that the results obtained for the early steps of A-90289 and sphaerimicin biosynthesis are also valid for the biosynthetic formation of muraymycins. Bioinformatic analyses of the biosynthetic gene clusters of A-90289 antibiotics, caprazamycins and muraymycins revealed six shared ORFs overall [128]. A sequence comparison of a range of transaldolases gave 47% identity and 78% similarity of Mur17 with LipK [129]. Overall, these insights suggest that the formation of the GlyU intermediate **101** and very likely also of the whole aminoribosylated nucleoside core structure occur in a conserved manner. Further studies on muraymycin biosynthesis are still pending.

## Conclusion

In summary, this review describes a promising class of antimicrobially active natural products, the uridine-derived muraymycins. Muraymycins are one subclass of nucleoside antibiotics inhibiting the membrane protein translocase I (MraY), a key enzyme in the intracellular part of peptidoglycan formation. Synthetic methodology for the preparation of muraymycins and their analogues has been established, and first SAR insights revealed that the design of structurally simplified, biologically active muraymycin analogues is an auspicious approach. However, further SAR studies as well as investigations on the interplay of target inhibition and cellular uptake for the antibiotic activity are surely desirable. Studies on muraymycin biosynthesis may not only be of academic interest, but could also lead to semi- or mutasynthetic methodology for the preparation of novel muraymycin analogues. Several laboratories around the world currently perform research on muraymycins and other uridine-derived nucleoside antibiotics. Hopefully, this work will contribute to the development of urgently needed novel antimicrobial drugs.

## References

[1] Fleming A (2001). Bull W H O.

[2] Chain E, Florey H W, Gardner A D, Heatley N G, Jennings M A, Orr-Ewing J, Sanders A G (1940). Lancet.

[3] Abraham E P, Chain E (1940). Nature.

[4] Kirby W M M (1944). Science.

[5] Levy S B, Marshall B (2004). Nat Med.

[6] Bush K (2012). Curr Opin Pharmacol.

[7] O'Connell K M G, Hodgkinson J T, Sore H F, Welch M, Salmond G P C, Spring D R (2013). Angew Chem, Int Ed.

[8] von Nussbaum F, Brands M, Hinzen B, Weigand S, Häbich D (2006). Angew Chem, Int Ed.

[9] Butler M S, Buss A D (2006). Biochem Pharmacol.

[10] Alekshun M N, Levy S B (2007). Cell.

[11] Walsh C (2000). Nature.

[12] Wright G D (2003). Curr Opin Chem Biol.

[13] Wright G D (1999). Curr Opin Microbiol.

[14] Katz L, Ashley G W (2005). Chem Rev.

[15] Li X-Z, Nikaido H (2009). Drugs.

[16] Denyer S P, Maillard J-Y (2002). J Appl Microbiol.

[17] Lambert P A (2002). J Appl Microbiol.

[18] Barrett J F (2004). Expert Opin Ther Targets.

[19] Otto M (2013). Int J Med Microbiol.

[20] Bonten M J M, Willems R, Weinstein R A (2001). Lancet Infect Dis.

[21] Walsh C (2003). Nat Rev Microbiol.

[22] McDonald L A, Barbieri L R, Carter G T, Lenoy E, Lotvin J, Petersen P J, Siegel M M, Singh G, Williamson R T (2002). J Am Chem Soc.

[23] Kimura K, Bugg T D H (2003). Nat Prod Rep.

[24] Winn M, Goss R J M, Kimura K, Bugg T D H (2010). Nat Prod Rep.

[25] Ichikawa S, Yamaguchi M, Matsuda A (2015). Curr Med Chem.

[26] Takatsuki A, Arima K, Tamura G (1971). J Antibiot.

[27] Takatsuki A, Tamura G (1971). J Antibiot.

[28] Takatsuki A, Tamura G (1971). J Antibiot.

[29] Eckardt K, Thrum H, Bradler G, Tonew E, Tonew M (1975). J Antibiot.

[30] Thrum H, Eckardt K, Bradler G, Fügner R, Tonew E, Tonew M (1975). J Antibiot.

[31] Eckardt K, Ihn W, Tresselt D, Krebs D (1981). J Antibiot.

[32] Vogel P, Petterson D S, Berry P H, Frahn J L, Anderton N, Cockrum P A, Edgar J A, Jago M V, Lanigan G W, Payne A L (1981). Aust J Exp Biol Med Sci.

[33] Seto H, Otake N, Sato S, Yamaguchi H, Takada K, Itoh M, Lu H S M, Clardy J (1988). Tetrahedron Lett.

[34] Yamaguchi H, Sato S, Yoshida S, Takada K, Itoh M, Seto H, Otake N (1986). J Antibiot.

[35] Ochi K, Ezaki M, Iwami M, Komori T, Kohsaka M (1990). FR-900493 substance, a process for its production and a pharmaceutical composition containing the same. U.S. Patent.

[36] Inukai M, Isono F, Takahashi S, Enokita R, Sakaida Y, Haneishi T (1989). J Antibiot.

[37] Isono F, Inukai M, Takahashi S, Haneishi T, Kinoshita T, Kuwano H (1989). J Antibiot.

[38] Isono F, Katayama T, Inukai M, Haneishi T (1989). J Antibiot.

[39] Karwowski J P, Jackson M, Theriault R J, Chen R H, Barlow G J, Maus M L (1989). J Antibiot.

[40] Chen R H, Buko A M, Whittern D N, McAlpine J B (1989). J Antibiot.

[41] Fernandes P B, Swanson R N, Hardy D J, Hanson C W, Coen L, Rasmussen R R, Chen R H (1989). J Antibiot.

[42] Chatterjee S, Nadkarni S R, Vijayakumar E K S, Patel M V, Ganguli B N, Fehlhaber H-W, Vertesy L (1994). J Antibiot.

[43] Xie Y, Chen R, Si S, Sun C, Xu H (2007). J Antibiot.

[44] Xie Y, Xu H, Si S, Sun C, Chen R (2008). J Antibiot.

[45] Isono K, Uramoto M, Kusakabe H, Kimura K-I, Izaki K, Nelson C C, McCloskey J A (1985). J Antibiot.

[46] Igarashi M, Nakagawa N, Doi N, Hattori S, Naganawa H, Hamada M (2003). J Antibiot.

[47] Igarashi M, Takahashi Y, Shitara T, Nakamura H, Naganawa H, Miyake T, Akamatsu Y (2005). J Antibiot.

[48] Naganawa H, Hamada M, Igarashi M, Takeuchi T (2001). Antibiotic caprazamycins and process for producing the same. Canadian Patent.

[49] Carter G T, Lotvin J A, McDonald L A (2003). Antibiotics AA-896. WO Patent.

[50] Bugg T D H, Walsh C T (1992). Nat Prod Rep.

[51] van Heijenoort J (2001). Nat Prod Rep.

[52] Vollmer W, Blanot D, De Pedro M A (2008). FEMS Microbiol Rev.

[53] Osborn M J (1969). Annu Rev Biochem.

[54] Barreteau H, Kovač A, Boniface A, Sova M, Gobec S, Blanot D (2008). FEMS Microbiol Rev.

[55] Bouhss A, Trunkfield A E, Bugg T D H, Mengin-Lecreulx D (2008). FEMS Microbiol Rev.

[56] Gautam A, Vyas R, Tewari R (2011). Crit Rev Biotechnol.

[57] Egan A J F, Vollmer W (2013). Ann N Y Acad Sci.

[58] Ikeda M, Wachi M, Jung H K, Ishino F, Matsuhashi M (1991). J Bacteriol.

[59] Boyle D S, Donachie W D (1998). J Bacteriol.

[60] Branstrom A A, Midha S, Longley C B, Han K, Baizman E R, Axelrod H R (2000). Anal Biochem.

[61] Barbosa M D F S, Ross H O, Hillman M C, Meade R P, Kurilla M G, Pompliano D L (2002). Anal Biochem.

[62] Thanassi J A, Hartman-Neumann S L, Dougherty T J, Dougherty B A, Pucci M J (2002). Nucleic Acids Res.

[63] Lara B, Mengin-Lecreulx D, Ayala J A, van Heijenoort J (2005). FEMS Microbiol Lett.

[64] Struve W G, Neuhaus F C (1965). Biochem Biophys Res Commun.

[65] Anderson J S, Matsuhashi M, Haskin M A, Strominger J L (1965). Proc Natl Acad Sci U S A.

[66] Heydanek M G, Struve W G, Neuhaus F C (1969). Biochemistry.

[67] Pless D D, Neuhaus F C (1973). J Biol Chem.

[68] Lloyd A J, Brandish P E, Gilbey A M, Bugg T D H (2004). J Bacteriol.

[69] Al-Dabbagh B, Henry X, Ghachi M E, Auger G, Blanot D, Parquet C, Mengin-Lecreulx D, Bouhss A (2008). Biochemistry.

[70] Bouhss A, Mengin-Lecreulx D, Le Beller D, van Heijenoort J (1999). Mol Microbiol.

[71] Chung B C, Zhao J, Gillespie R A, Kwon D-Y, Guan Z, Hong J, Zhou P, Lee S-Y (2013). Science.

[72] Bernhardt T G, Roof W D, Young R (2000). Proc Natl Acad Sci U S A.

[73] Bernhardt T G, Struck D K, Young R (2001). J Biol Chem.

[74] Rodolis M T, Mihalyi A, O'Reilly A, Slikas J, Roper D I, Hancock R E W, Bugg T D H (2014). ChemBioChem.

[75] Andrews J M (2001). J Antimicrob Chemother.

[76] Yamashita A, Norton E, Petersen P J, Rasmussen B A, Singh G, Yang Y, Mansour T S, Ho D M (2003). Bioorg Med Chem Lett.

[77] Tanino T, Al-Dabbagh B, Mengin-Lecreulx D, Bouhss A, Oyama H, Ichikawa S, Matsuda A (2011). J Med Chem.

[78] Spork A P, Büschleb M, Ries O, Wiegmann D, Boettcher S, Mihalyi A, Bugg T D H, Ducho C (2014). Chem – Eur J.

[79] Brandish P E, Burnham M K, Lonsdale J T, Southgate R, Inukai M, Bugg T D H (1996). J Biol Chem.

[80] Brandish P E, Kimura K, Inukai M, Southgate R, Lonsdale J T, Bugg T D (1996). Antimicrob Agents Chemother.

[81] Bouhss A, Crouvoisier M, Blanot D, Mengin-Lecreulx D (2004). J Biol Chem.

[82] Fer M J, Bouhss A, Patrão M, Le Corre L, Pietrancosta N, Amoroso A, Joris B, Mengin-Lecreulx D, Calvet-Vitale S, Gravier-Pelletier C (2015). Org Biomol Chem.

[83] Shapiro A B, Jahić H, Gao N, Hajec L, Rivin O (2012). J Biomol Screening.

[84] Mengin-Lecreulx D, Parquet C, Desviat L R, Plá J, Flouret B, Ayala J A, van Heijenoort J (1989). J Bacteriol.

[85] Ma Y, Münch D, Schneider T, Sahl H-G, Bouhss A, Ghoshdastider U, Wang J, Dötsch V, Wang X, Bernhard F (2011). J Biol Chem.

[86] Lin Y-I, Li Z, Francisco G D, McDonald L A, Davis R A, Singh G, Yang Y, Mansour T S (2002). Bioorg Med Chem Lett.

[87] Zhu X-F, Williams H J, Scott A I (2000). J Chem Soc, Perkin Trans 1.

[88] Myers A G, Gin D Y, Rogers D H (1994). J Am Chem Soc.

[89] Banfi L, Cardani S, Potenza D, Scolastico C (1987). Tetrahedron.

[90] Hirano S, Ichikawa S, Matsuda A (2005). Angew Chem, Int Ed.

[91] Hirano S, Ichikawa S, Matsuda A (2007). J Org Chem.

[92] Hirano S, Ichikawa S, Matsuda A (2008). J Org Chem.

[93] Kimura J, Kobayashi H, Miyahara O, Mitsunobu O (1986). Bull Chem Soc Jpn.

[94] Tao B, Schlingloff G, Sharpless K B (1998). Tetrahedron Lett.

[95] Ichikawa S, Hayashi R, Hirano S, Matsuda A (2008). Org Lett.

[96] Tanino T, Hirano S, Ichikawa S, Matsuda A (2008). Nucleic Acids Symp Ser.

[97] Tanino T, Ichikawa S, Shiro M, Matsuda A (2010). J Org Chem.

[98] Aleiwi B A, Schneider C M, Kurosu M (2012). J Org Chem.

[99] Spork A P, Koppermann S, Dittrich B, Herbst-Irmer R, Ducho C (2010). Tetrahedron: Asymmetry.

[100] Aggarwal V K, Ford J G, Thompson A, Jones R V H, Standen M C H (1996). J Am Chem Soc.

[101] Aggarwal V K, Harvey J N, Richardson J (2002). J Am Chem Soc.

[102] Spork A P, Koppermann S, Ducho C (2009). Synlett.

[103] Sarabia F, Martín-Ortiz L, López-Herrera F J (2003). Org Lett.

[104] Sarabia F, Martín-Ortiz L (2005). Tetrahedron.

[105] Spork A P, Ducho C (2013). Synlett.

[106] Dondoni A, Perrone D (2000). Org Synth.

[107] Ries O, Büschleb M, Granitzka M, Stalke D, Ducho C (2014). Beilstein J Org Chem.

[108] Laïb T, Chastanet J, Zhu J (1998). J Org Chem.

[109] Spork A P, Ducho C (2010). Org Biomol Chem.

[110] Spork A P, Wiegmann D, Granitzka M, Stalke D, Ducho C (2011). J Org Chem.

[111] Schmidt U, Lieberknecht A, Schanbacher U, Beuttler T, Wild J (1982). Angew Chem, Int Ed.

[112] Burk M J (1991). J Am Chem Soc.

[113] Masquelin T, Broger E, Müller K, Schmid R, Obrecht D (1994). Helv Chim Acta.

[114] Tanino T, Ichikawa S, Al-Dabbagh B, Bouhss A, Oyama H, Matsuda A (2010). ACS Med Chem Lett.

[115] Takeoka Y, Tanino T, Sekiguchi M, Yonezawa S, Sakagami M, Takahashi F, Togame H, Tanaka Y, Takemoto H, Ichikawa S (2014). ACS Med Chem Lett.

[116] Schmidtgall B, Spork A P, Wachowius F, Höbartner C, Ducho C (2014). Chem Commun.

[117] Schmidtgall B, Höbartner C, Ducho C (2015). Beilstein J Org Chem.

[118] Cheng L, Chen W, Zhai L, Xu D, Huang T, Lin S, Zhou X, Deng Z (2011). Mol BioSyst.

[119] Yin X, Zabriskie T M (2004). ChemBioChem.

[120] Yin X, McPhail K L, Kim K, Zabriskie T M (2004). ChemBioChem.

[121] Ju J, Ozanick S G, Shen B, Thomas M G (2004). ChemBioChem.

[122] Hamed R B, Gomez-Castellanos J R, Henry L, Ducho C, McDonough M A, Schofield C J (2013). Nat Prod Rep.

[123] Lemke A, Büschleb M, Ducho C (2010). Tetrahedron.

[124] Lemke A, Ducho C (2016). Eur J Org Chem.

[125] Büschleb M, Granitzka M, Stalke D, Ducho C (2012). Amino Acids.

[126] Chi X, Pahari P, Nonaka K, Van Lanen S G (2011). J Am Chem Soc.

[127] Yang Z, Chi X, Funabashi M, Baba S, Nonaka K, Pahari P, Unrine J, Jacobsen J M, Elliott G I, Rohr J (2011). J Biol Chem.

[128] Barnard-Britson S, Chi X, Nonaka K, Spork A P, Tibrewal N, Goswami A, Pahari P, Ducho C, Rohr J, Van Lanen S G (2012). J Am Chem Soc.

[129] Funabashi M, Baba S, Takatsu T, Kizuka M, Ohata Y, Tanaka M, Nonaka K, Spork A P, Ducho C, Chen W-C L (2013). Angew Chem, Int Ed.

